# Genetic Dissection of Seedling Root System Architectural Traits in a Diverse Panel of Hexaploid Wheat through Multi-Locus Genome-Wide Association Mapping for Improving Drought Tolerance

**DOI:** 10.3390/ijms22137188

**Published:** 2021-07-02

**Authors:** Thippeswamy Danakumara, Jyoti Kumari, Amit Kumar Singh, Subodh Kumar Sinha, Anjan Kumar Pradhan, Shivani Sharma, Shailendra Kumar Jha, Ruchi Bansal, Sundeep Kumar, Girish Kumar Jha, Mahesh C. Yadav, P.V. Vara Prasad

**Affiliations:** 1 Division of Genetics, Indian Council of Agricultural Research (ICAR)—Indian Agricultural Research Institute, New Delhi 110012, India; danakumarat@gmail.com (T.D.); jhashail78@gmail.com (S.K.J.); 2 Division of Germplasm Evaluation, ICAR-National Bureau of Plant Genetic Resources, New Delhi 110012, India; shivani13biotech@gmail.com (S.S.); ruchi.bansal@icar.gov.in (R.B.); 3 Division of Genomic Resources, ICAR-National Bureau of Plant Genetic Resources, New Delhi 110012, India; amit.singh5@icar.gov.in (A.K.S.); akkumaranjan@gmail.com (A.K.P.); sundeepksharma77@rediffmail.com (S.K.); mahesh.yadav1@icar.gov.in (M.C.Y.); 4 ICAR-National Institute of Plant Biotechnology, New Delhi 110012, India; subodh.sinha@icar.gov.in; 5 Division of Agricultural Economics, ICAR-Indian Agricultural Research Institute, New Delhi 110012, India; girish.stat@gmail.com; 6 Department of Agronomy, Kansas State University, Manhattan, KS 66506, USA; vara@ksu.edu

**Keywords:** wheat, germplasm, root traits, root architecture, quantitative trait nucleotides, ML-GWAS

## Abstract

Cultivars with efficient root systems play a major role in enhancing resource use efficiency, particularly water absorption, and thus in drought tolerance. In this study, a diverse wheat association panel of 136 wheat accessions including mini core subset was genotyped using Axiom 35k Breeders’ Array to identify genomic regions associated with seedling stage root architecture and shoot traits using multi-locus genome-wide association studies (ML-GWAS). The association panel revealed a wide variation of 1.5- to 50-fold and were grouped into six clusters based on 15 traits. Six different ML-GWAS models revealed 456 significant quantitative trait nucleotides (QTNs) for various traits with phenotypic variance in the range of 0.12–38.60%. Of these, 87 QTNs were repeatedly detected by two or more models and were considered reliable genomic regions for the respective traits. Among these QTNs, eleven were associated with average diameter and nine each for second order lateral root number (SOLRN), root volume (RV) and root length density (RLD). A total of eleven genomic regions were pleiotropic and each controlled two or three traits. Some important candidate genes such as Formin homology 1, Ubiquitin-like domain superfamily and ATP-dependent 6-phosphofructokinase were identified from the associated genomic regions. The genomic regions/genes identified in this study could potentially be targeted for improving root traits and drought tolerance in wheat.

## 1. Introduction

Globally, wheat (*Triticum aestivum* L.) is one of the most important cultivated food grain crops with an area and production of 218.54 million hectares and 771.71 million tons, respectively [1]. Wheat ranks second after rice (*Oryza sativa* L.) as a major staple food crop in India with an acreage of 30.55 million hectares, and total production and productivity of 107.18 million tons and 3508 kg/ha, respectively [2]. Wheat is adapted to various climatic conditions including areas vulnerable to heat, salt and drought stress [3,4]. Drought is one of the major challenges that limits crop growth and yield in different areas of the world [5,6]. Drought stress can cause significant yield reduction in wheat and its impact varies with intensity, timing and duration of stress relative to crop growth stages. Yield reduction to the tune of 21.0%, 25.8% and 32.0% were reported in wheat under mild (55–70% relative soil water content), moderate (35–55%, relative soil water content) and severe (<35% relative soil water content) drought stress, respectively [7].

Root traits are important not only for water absorption from a drought stress perspective, but also for soil nutrient uptake and environmental stress tolerance [8,9,10]. Morphology of the root system includes basic features such as root length, root length density, root diameter, surface area and volume which influence the root structure’s spatial arrangement and are significantly correlated with water uptake and nutrient absorption [11,12,13]. Drought tolerance is directly associated with root diameter, as thicker roots with large xylem vessels are more effective towards extraction of water and nutrients from deep soil layers under rainfed condition [12]. Fine roots and the number of root tips, the major components of root systems, often increase the water and nutrient uptake efficiency by increasing the root surface area and volume [8,10]. Further, deep rooting, an essential root feature allowing access to water from deeper soil profiles, improves crop productivity [14]. Therefore, one approach to improve drought tolerance is to identify and introgress deeper rooting alleles in shallow rooted and drought prone cultivars [15,16,17]. Thus, the study of root system architecture (RSA) features can help define proxy traits to enhance tolerance to various soil types and status of moisture and nutrient stress conditions [18]. RSA characteristics are governed by polygenes with additive cumulative gene action and are influenced by the environment [12]. Due to the difficulty of high-throughput phenotyping of RSA characteristics in field conditions, their optimization was ignored [10,12,19]. However, seedling level root architecture is associated with adult plant root architecture and is being studied to translate the knowledge for later growth stages [20,21].

The knowledge on extent of genetic variability in wheat germplasm collections could pave the way for successful conservation and utilization in crop improvement. In recent years, cost effective advanced high throughput genotyping approaches, such as single nucleotide polymorphism (SNP) arrays and sequencing based genotyping methods such as genotyping by sequencing (GBS), restricted site associated DNA sequencing (RAD-seq) and whole genome re-sequencing, have greatly accelerated genomic level characterization of genetic diversity of crop germplasm resources in many species. Availability of these techniques has made it feasible to conduct whole genome scan studies, such as association mapping (AM) and genomic selection in a variety of crops including wheat, maize (*Zea mays* L.), and rice [22,23]. Association mapping is an alternative approach to mapping quantitative trait loci (QTLs) controlling complex traits which is based on the principle of linkage disequilibrium between marker and trait. In contrast to the conventional QTL approach, the AM approach enables screening of a large number of alleles present in natural populations of species, thus providing greater resolution mapping of a target trait. In the past couple of years, AM has been widely applied to map traits in many crop species including wheat, rice, barley (*Hordeum vulgare* L.), maize and soybean (*Glycine max* L. Merril) [21,24,25,26,27] with the availability of high-throughput genotyping and phenotyping platforms [22,23].

Exploring genetic variability of root traits in a diverse germplasm collection could assist in development of varieties with desired root features for drought tolerance or target environments. To date, only a few reports are available on genetic variability and QTL mapping of root traits in wheat [28,29,30]. The wheat core and mini core collections constituted from the large wheat accessions conserved in the national and international gene banks could be a valuable genetic resource for mapping complex traits using a genome wide association study (GWAS). The GWAS could facilitate uncovering genomic regions/genes controlling various root architecture traits. In our earlier study, a core set of wheat genotypes was identified from the entire wheat collection in the national gene bank of India based on 35 agro-morphological traits [31]. This core set was subsequently reduced to a mini core collection that captured huge variability for various traits and could be used for mapping root associated traits. The AM analysis on this mini core collection could facilitate identification of genes/genomic regions associated with RSA traits that could be exploited in a breeding program to develop drought tolerant cultivars. Keeping this in mind, the objectives of this research were: (1) to study genetic variation of seedling root system architecture in a diverse set of wheat genotypes including a subset of wheat mini core collection; and (2) to identify genomic regions/candidate regions linked with these traits using association mapping.

## 2. Results

### 2.1. Phenotypic Analyses

There were large differences for various root traits (Table 1) in the wheat association panel owing to their diverse geographical origin (Supplementary Table S1). The abbreviations used here are listed and expanded in Table 1. A significant difference (*p* ≤ 0.001) was observed among the genotypes for each phenotypic trait except for lateral root density, LRD (Supplementary Table S2). Mean values of the traits (mean ± SE) were as follows: total root size, TRS (242.94 ± 5.35); first order lateral root number, FOLRN (95.22 ± 3.58); second order lateral root number, SOLRN (165.57 ± 8.97); lateral root size, LRS (0.39 ± 0.01); lateral root density, LRD (2.81 ± 0.08); root length density, RLD (0.49 ± 0.01); average diameter, AD (0.38 ± 0.01); root volume, RV (0.16 ± 0.01); seminal root number, SRN (5.06 ± 0.04); shoot dry weight, SDW (35.58 ± 0.88); root dry weight, RDW (10.76 ± 0.32); shoot length, SL (30.61 ± 0.41); root length, RL (24.81 ± 0.54); root shoot dry weight ratio, RSDWR (0.31 ± 0.01) and specific root length, SRL (2.52 ± 0.07) (Table 1). We observed a high range of coefficient variation (%CV) for all the phenotypic traits. The highest CV was observed for SOLRN (64.10%) followed by FOLRN (44.50%) and LRS (38.39%). The lowest CV was observed for AD (8.46%) followed by SRN (10.29%). The estimates of broad-sense heritability were low to moderately high, ranging from 15.16% for LRD, to 78.95% for AD (Supplementary Table S2).

The frequency distribution pattern of traits is presented in Supplementary Figure S1. Most of the traits followed normal distribution, except SRN and SL which were negatively skewed, while SOLRN and SRL were positively skewed. Selected wheat genotypes with high and low mean performance of root and shoot morphological traits are shown in Supplementary Table S3. The wheat genotype IC28755 exhibited maximum root length of 37.50 cm, whereas the genotype EC6903, a collection from the USA, showed a maximum shoot length of 40.76 cm. The genotype IC128151 (C306) had highest value of 60.41 mg for SDW and the genotype EC576889 had maximum value for RDW at 23.06 mg. The trait root-shoot dry weight ratio indicated the amount of root dry mass to shoot dry mass. The genotype IC532019 had the highest ratio of 0.59 contributing higher root dry mass than shoot at seedling stage. Donors for multiple traits were also identified. Genotype IC82425A exhibited the highest mean values for SOLRN, LRS and RV. Further the genotype IC128151 (C306) was among the top ten genotypes for seven traits, namely TRS, FOLRN, LRD, RLD, RV, SDW and RDW. The genotype IC542076 ranked in the top ten genotypes for seven traits: TRS, SOLRN, RLD, RV, SRN, SDW and RDW, and was therefore considered promising for tolerating drought by extracting water stored in the deeper soil layers. The genotypes IC128151, IC82425A and IC542076 were superior for several root and shoot traits in the drought experiment carried out at the seedling stage under pot screening during the winter season 2019–2020(data not presented). In addition, genotypes with low mean performance were also identified so they could be utilized to generate bi-parental mapping populations or multiparent advanced generation intercross (MAGIC) populations. An exotic collection from the USA, EC339632, was observed to have lowest mean performance for TRS, FOLRN, SOLRN, RLD, RV, SRN, RDW and RL, whereas EC187159 showed lower mean values for TRS, SOLRN, LRS, RLD, SRN, SDW, RDW and SL.

### 2.2. Multivariate Analyses of Phenotypic Traits

#### 2.2.1. Association Analysis

**Figure 1:**
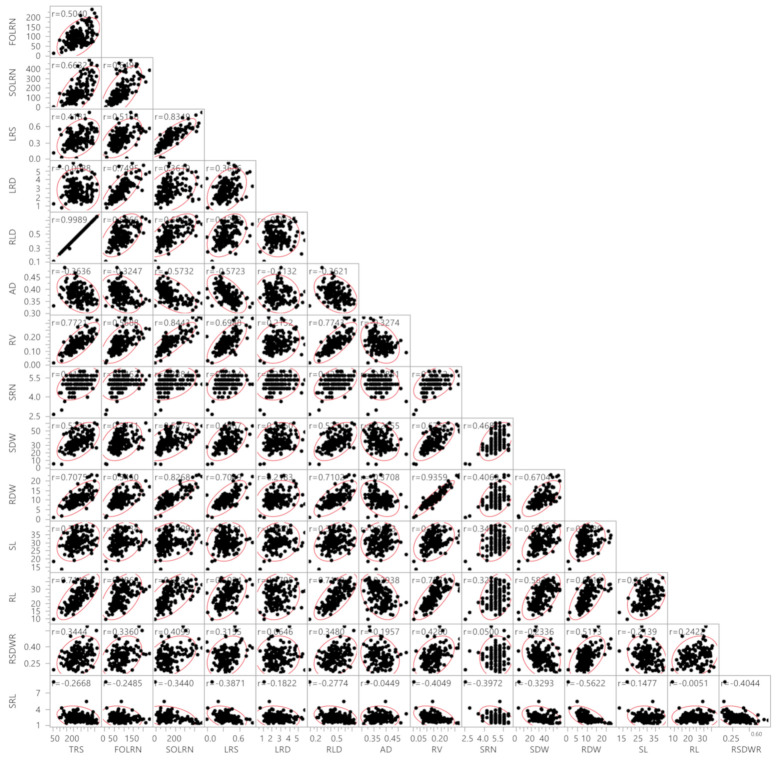
Correlations between root system architecture traits and shoot traits. TRS, total root size (cm); FOLRN, first order lateral root number; SOLRN, second order lateral root number; LRS, lateral root size; LRD, lateral root density (cm^−1^); RLD, root length density cm^−2^; AD, average diameter (mm); RV, root volume (cm^3^); SRN, seminal root number; SDW, shoot dry weight (mg); RDW, root dry weight (mg); SL, shoot length (cm); RL, root length (cm); RSDWR, root shoot dry weight ratio; SRL, specific root length (cm mg^−1^). Red boundary for each pairwise correlation displays density ellipses covering 95% points between each variable. The density ellipses are a graphical indicator of the correlation between two variables. It collapses diagonally as the correlation between the two variables approaches either 1 or −1. It is more circular if the two variables are less correlated.

Association analysis revealed the influence of a correlated response from different traits (Figure 1). A highly significant positive association was observed between TRS with FOLRN (r = 0.50), SOLRN (r = 0.65), LRS (r = 0.42), RLD (r = 0.99), RV (r = 0.77), SRN (r = 0.42), SDW (r = 0.52), RDW (r = 0.70) and RL (r = 0.71). TRS showed a negative association with AD (r = −0.37), SRL (−0.27) and LRD (r = −0.01). The traits FOLRN, SOLRN, LRS and RV had a highly significant positive association with all traits except AD and SRL. Further, LRD showed a highly significant positive correlation with FOLRN (r = 0.75), SOLRN (r = 0.36) and LRS (r = 0.36) and a positive significant correlation with RV (r = 0.21), SRN (r = 0.23), SDW (r = 0.20) and RDW (r = 0.21). The RLD had a positive and highly significant association with all traits, while there were negative associations with AD (r = −0.37), LRD (r = −0.01) and SRL (r = −0.27). In contrast, AD was significantly correlated with all the traits in a negative direction except SL. Similarly, SRL was also negatively correlated with all traits.

#### 2.2.2. Principal Component Analysis

Principal component analysis (PCA) was performed for 15 RSA and shoot traits to determine their contribution to overall variability. The first four principal components (PCs) in the PCA analysis with eigenvalues >1 contributed a cumulative variance of 79.24% among the genotypes (Supplementary Table S4). The scree plot showing the eigenvalues on the *y*-axis and the number of components on the *x*-axis also depicted the number of relevant PCs to explain maximum variance (Supplementary Figure S2A). Based on the contribution towards the principal components, RV, RDW, SOLRN, RLD, TRS, RL, LRS, FOLRN, SRN and RSDWR were the major contributors toward PC1 and were most significantly associated with the genotypic variations. Likewise, SDW and SL were major contributors to PC2, LRD and FOLRN to PC3 and SRL to PC4 (Supplementary Table S4). Length of coordinates or distance from the origin revealed the amount of variation contributed by each trait (Supplementary Figure S2B) which also ascertained about their major contributions. The PCA also indicated trait association, as the correlated traits have fewer angles between them or are mostly parallel to each other. In this case, the positive quadrant had correlated traits SDW and SRN. The results indicated that AD and SRL were opposite to other traits in the second quadrant, hence their negative association with them.

#### 2.2.3. Clustering of Genotypes

The diverse association panel of the wheat mini core subset (136 accessions, four had a poor SNP call and were therefore excluded from the analysis) was divided into six clusters (Supplementary Figure S3) when subjected to hierarchical analysis based on the 15 root and shoot traits, which showed that the wheat genotypes were sufficiently diverse. Among different classes, the largest cluster (II) contained 38 member genotypes and the smallest cluster (III) had 2 members (EC339632, a USDA collection and EC578153 and a synthetic line). Clusters six and three consisted of genotypes with high and low performance respectively for many traits such as TRS, FOLRN and SOLRN (Supplementary Table S5). A heatmap generated by two-way clustering also depicted that cluster six had high performing genotypes, whereas cluster 1 had low performing genotypes for many of the traits (Supplementary Figure S3).

### 2.3. Marker Distribution, Linkage Distribution and Population Structure

The SNP genotyping of 136 wheat genotypes yielded a total of 16,616 polymorphic SNPs with exact chromosomal position and minor allele frequency (MAF) ≥ 10. The SNP markers were proportionally distributed across the 21 chromosomes and their number ranged from 287 for Chr4D to 1179 for Chr2B, and the average SNP markers per chromosome was 791. The polymorphic SNPs covered 14066.28 Mb of wheat genome. The sub-genome A included 5355 SNPs, the sub-genome B had 6343 SNPs and 4918 SNPs were located on the sub-genome D. On sub-genome A, maximum SNPs were found on 2A (952) followed by 1A (911). Further, on sub-genome B, maximum SNPs were found on Chr2B (1178), followed by Chr5B (1125). Chr2D of Sub-genome D had maximum SNPs (1037) followed by Chr1D (895) (Table 2). The SNP marker density per chromosome per Mb ranged from 0.56 for 4D to 1.59 for 2D, with a mean of 1.182. These polymorphic 16,616 SNP markers were used to estimate genome-wide linkage disequilibrium (LD) and the pairwise pattern of LD was calculated using r^2^ of allele pairs between two loci. Among these, a total of 19,528 SNP pairs were in perfect LD (r^2^ = 1), with B sub-genome having a maximum (7287) number followed by D (7100) and A (5141) sub-genomes. Overall, in the current association panel, low mean LD was observed across the 21 chromosomes with values ranging from 0.087 (4D) to 0.298 (1D). The average LD (r^2^) per chromosome ranged from 0.087 for 4D to 0.298 for 1D. The measures of LD decay for all 21 chromosomes are summarized in Supplementary Figure S4. The LD decay in the AM panel was determined at r^2^ = 0.157 (background LD). LD decayed the fastest in sub-genome A followed by sub-genome D and B. In sub-genome A, LD decayed at 3 Mb compared to 3.4 Mb in sub-genome B and 4.2 Mb in sub-genome D (Table 2 and Supplementary Figure S4).

**Figure 2:**
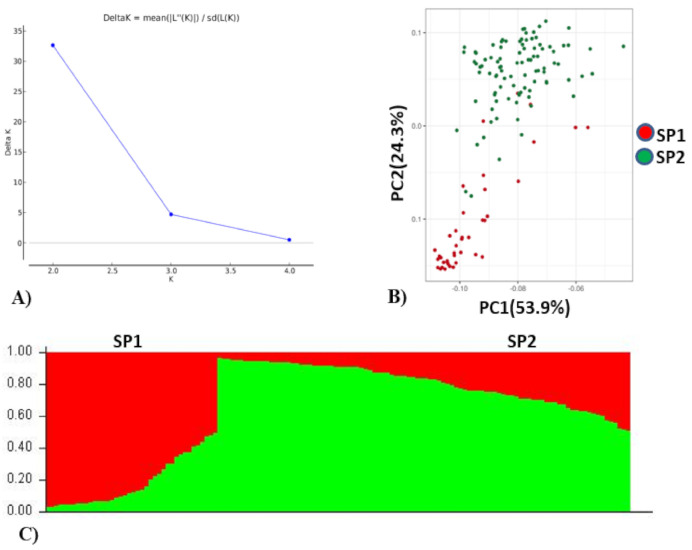
Population stratification of wheat association mapping panel. (**A**) Delta K, rate of change from 2 to 4. (**B**) Biplot of principal component analysis (PCA), PCA 1 and PCA 2. (**C**) Bar plot of the AM panel showing the structuring of two subpopulations (SP1 and SP2) in different colors, *viz.* SP1 (red, 40 genotypes), SP2 (green, 96 genotypes).

Population analysis of the association panel was performed using both STRUCTURE and PCA which indicated optimal number of population (K) as two (Figure 2). Of the total accessions, 40 accessions belonged to subpopulation 1 and 96 accessions belonged to subpopulation 2. Further, in subpopulation 1 (SP1), 30.50% of the accessions were admixtures and the remaining 62.50% of the genotypes were pure (>80% similarity) which included 25% EC (exotic) collection and 75% IC (indigenous) collection. The IC belonged to multiple states in India (Rajasthan, Gujarat, Haryana, West Bengal, Uttarakhand, Uttar Pradesh, Himachal Pradesh, Madhya Pradesh and Maharashtra). The EC belonged to Israel, China, the USA and Australia. In subpopulation 2 (SP2), 42.70% of the genotypes were admixtures and 57.29% of the genotypes were pure which included 31.25% EC and 68.75% IC collection. The EC mainly belonged to Mexico, Russia, Finland and the USA. Based on PCA analysis, genotypes contributing more variation to the PC1 were parallel to the PC1 axis and genotypes contributing more variation to the PC2 were parallel to the PC2 axis (Figure 2). A total of 88.2% cumulative variance were explained by two PCs. It also indicated that genotypes used in this study were divided into two structured subpopulations.

### 2.4. Genome-Wide Association Studies

**Figure 3:**
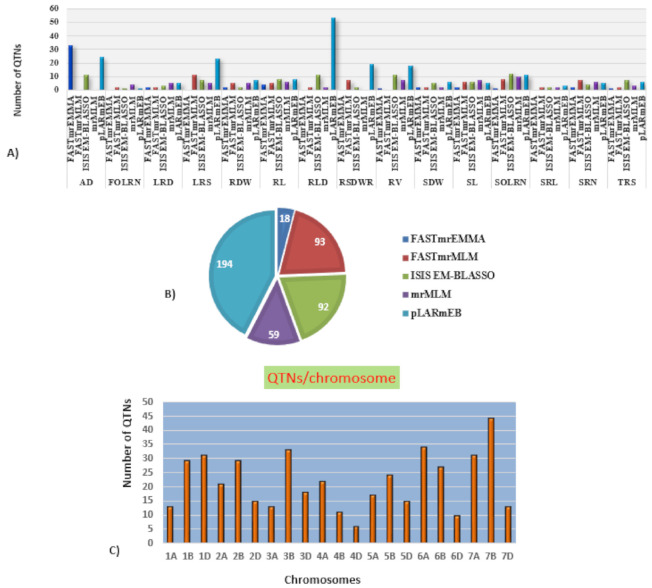
Distribution of the QTNs (quantitative trait nucleotides) identified using five different multi-locus GWAS (genome wide association study) models. (**A**) Number of significant QTNs detected for 15 traits across five multi-locus GWAS methods. (**B**) Number of significant QTNs detected using each of five multi-locus GWAS methods. (**C**) Number of QTNs per chromosome.

**Figure 4:**
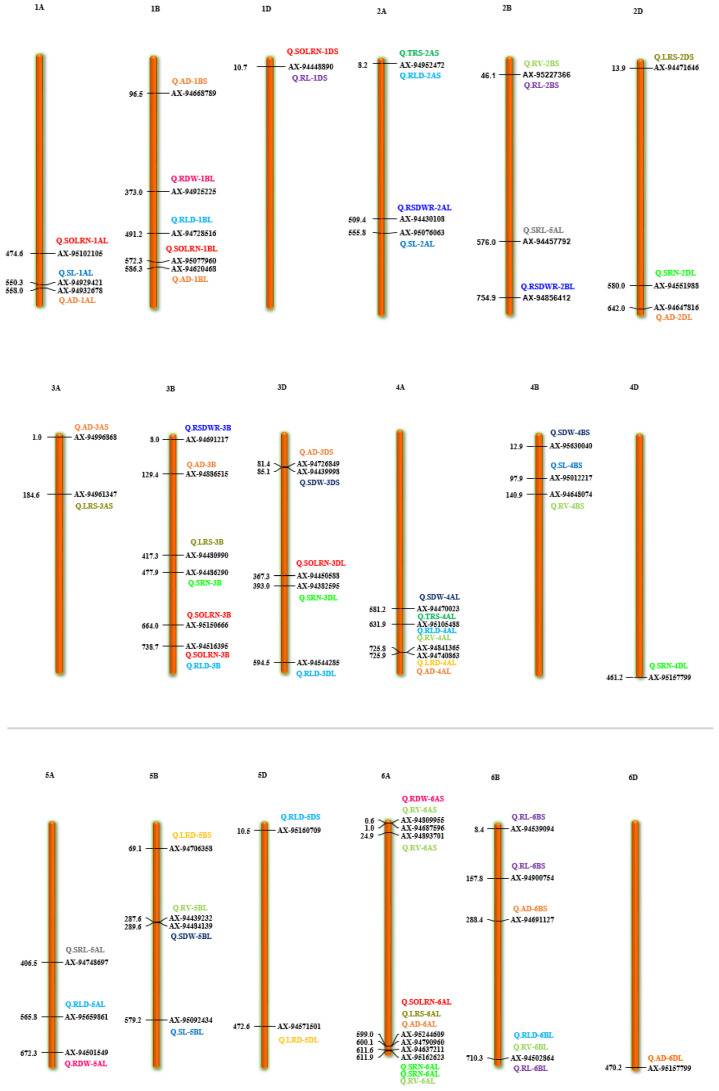

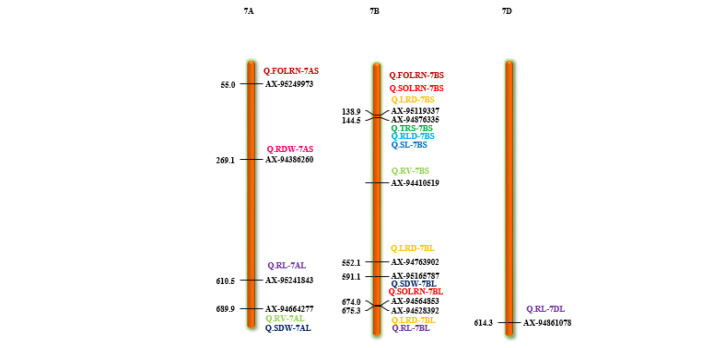
Chromosome location of 87 reliable QTNs (quantitative trait nucleotides) for 15 root and shoot traits. The color bars represent QTNs identified for different traits, red = SOLRN (second order lateral root number); dark green = TRS; (total root size) light green = RV (root volume); orange = AD (average diameter); pink = RDW (root dry weight); light blue = RLD (root length density); blue = SL (shoot length). (For interpretation of color references in this figure legend, refer to the web version of this article).

Marker-trait association analyses using six different ML-GWAS models (mrMLM, FASTmrMLM, FASTmrEMMA, pLARmEB, ISIS EM-BLASSO and pKWmEB) revealed 456 significant quantitative trait nucleotides (QTNs) for 15 seedling stage root architecture and shoot traits at a logarithm of the odd (LOD) score ≥3 with phenotypic variance in the range of 0.12–38.60%. Among these, 18, 59, 93, 92 and 194 significant QTNs were detected using FASTmrEMMA, mrMLM, FASTmrMLM, ISIS EM-BLASSO and pLARmEB, respectively (Table 3 and Figure 3). In this case, pKWmEB model could not detect any significant QTN. Maximum number of QTNs were recorded for AD (68) and RLD (68), followed by LRS (46), SOLRN (42) and RV (37). The extent of phenotypic variation of the QTNs varied from R^2^ = 1.8% for *QTN-SOLRN* at Chr 6A- AX-95244609 to R^2^ = 38.6% for *QTN-SOLRN* at Chr 3B- AX-95150666. The highest number of QTNs was identified in chromosome 7B (44) followed by 6A (34), 3B (33), 7A (31), 1D (31), 2B (29), 1B (29) and 6B (27). Further, among the total 456 QTNs, 87 QTNs and 27 QTNs were repeatedly detected by two, three or more ML-GWAS models, respectively. These were considered reliable QTNs for the respective traits. The reliable QTN that explained phenotypic variation ≥ 10% for the trait in at least one of the GWAS models was considered a major QTN. Among these 87 reliable QTNs, 11 QTNs were found to be associated with AD and 9 each for SOLRN, RV and RL, respectively. Further, a total of 12 QTNs for traits including SOLRN: AX-95077960, AX-94448890, AX-95150666 and AX-95244609; LRS: AX-95123855; AD: AX-95244609; RV: AX-94439232 and AX-94664277; SRN: AX-94551988; SDW: AX-94470023, RL: AX-94502864 and SRL: AX-94457792 were detected in four or five models (Table 3). Manhattan plots depicting identification of a reliable QTN for SDW (*Q.SDW-4AL*) and RL (*Q.RL-6BL*) by five ML-GWAS methods are presented in Supplementary Figures S5 and S6. Further, graphical representation of the genetic position of identified reliable QTNs on a physical map was given in Figure 4. It was revealed that the top five genomic regions that contained a maximum number of significant QTNs were found on chromosomes Chr1B, Chr3B, Chr4A, Chr6A and Chr7B.

#### 2.4.1. Colocalization of Root Architecture Loci

Different root traits are mostly correlated and complex biological mechanisms might be involved in coordination for their expression. The pleiotropic action of genetic loci on different traits or their tight linkage results in a correlation between traits. A total of 11 QTNs were found to be pleiotropic markers (Table 4). A locus on 1D (AX-94448890) was associated with root morphology (SOLRN and RL) and another locus on 2A (AX-94952472) was associated with TRS and RLD. Similarly, other loci were also identified as pleiotropic on 2B (AX-95227366) for RV and RL, 3B (AX-94516395) for SOLRN and RLD, 4A (AX-95105488) for TRS, RLD and RV, 6A (AX-95244609) for SOLRN, LRS, AD and 6B (X-94502864) for RLD, RV and RL. We also identified two pleiotropic loci on the short arm of 7B (AX-95123855) associated with root morphology traits (FOLRN, SOLRN, LRS) and QTN-7B-AX-95119337 associated with TRS and RLD whereas its long arm had a locus (AX-94528392) associated with RL and LRS. Other pleiotropic QTNs were 1DS- AX-94448890 (SOLRN and RL) and 7AL- AX-94664277 (RV and SDW) (Figure 4 and Table 4). These identified loci influencing several traits could be potential markers for marker-assisted breeding after validation.

#### 2.4.2. Allelic Effects of Identified Genomic Regions on Respective Phenotypes

**Figure 5:**
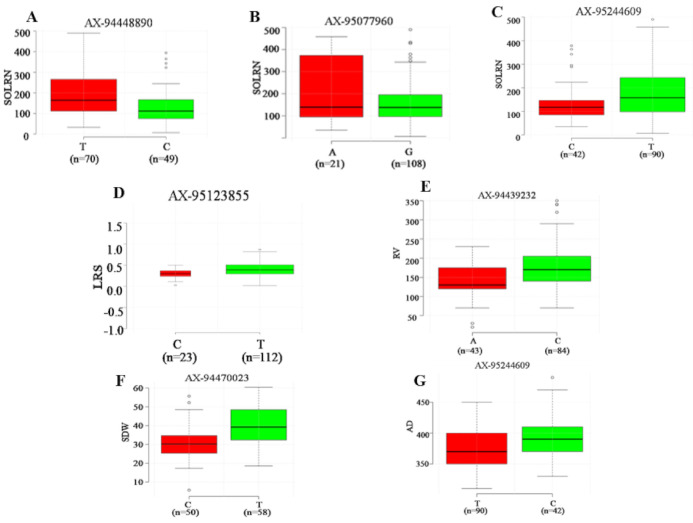
Boxplot for seven reliable QTNs with significant effects (*p* < 0.01) on corresponding root and shoot traits. For each QTNs, the germplasm lines were divided into two groups according to superior and inferior allele type. The *x*-axis represents the two alleles for each QTNs, while the *y*-axis corresponds to phenotypic value. Subfigures, (**A**–**C**) represent allelic differences for SOLRN and superior alleles for QTNs *Q.SOLRN-1DS, Q.SOLRN-1BL* and *Q.SOLRN-6AL* were T, A and T respectively. Subfigures (**D**–**G**) represent allelic differences for LRS, RV, SDW and AD for QTNs *Q.LRS-7BS, Q.RV-5BL, Q.SDW-4AL* and *Q.AD-6AL* with superor alleles as T, C, T and C respectively.

Seven major loci detected in four or more ML-GWAS methods were analyzed to determine the range of phenotypic variations for all traits (Figure 5). Association panel genotypes were divided into two classes according to allele types. It was observed that all seven QTNs demonstrated a significant effect on respective traits (*p* ≤ 0.01). Among these seven QTNs, three showed a significant effect on SOLRN (*Q.SOLRN-1DS*: AX-94448890, *Q.SOLRN-1BL*: AX-95077960, *Q.SOLRN-6AL*: AX-95244609) and one each on LRS (*Q.LRS-7BS*: AX-95123855), RV (*Q.RV-5BL*: AX-94439232), SDW (*Q.SDW-4AL*: AX-94470023) and AD (*Q.AD-6AL*: AX-95244609). The marker, AX-95244609 at Chr6AL was pleiotropic for SOLRN, LRS and AD. These significant associations suggest their plausible role in determining root architecture traits.

#### 2.4.3. Genes Linked to Quantitative Trait Nucleotides

Out of the total SNPs associated with various root and shoot traits, 52 SNP markers were found to be located within genes (Supplementary Table S6). Some important proteins encoded by these genes included glycoside hydrolase superfamily protein, carbonic anhydrase superfamily protein, auxin response factor, nitrogen regulatory protein, transmembrane protein, enhancer of polycomb-like protein and glutamate receptor. Moreover, we found that some of the loci corresponding to large effect QTNs and detected by multiple methods harbored genes for the growth and development of wheat root and shoot organs. For instance, QTN locus Chr2B: AX-94457792 for SRL is comprised of genes *TraesCS2B02G406800* and is predicted to encode Formin-like protein which regulates root-hair elongation in rice [32]. This locus also controls the actin cytoskeleton in root hair in wheat [33]. The other largely effected QTN associated with SL (Chr7BS: AX-94876335) was linked to a transcriptional responses of maize seedling root to phosphorus starvation [34]. Most QTNs associated with SRN were at loci containing genes predicted to encode Histone-lysine N-methyltransferase ATXR2, ATPase, F1/V1/A1 complex, alpha/beta subunit and the N-terminal domain superfamily. Other harboring genes encode nucleotide-diphospho-sugar transferases, ribosome-inactivating protein superfamily and papain-like cysteine peptidase superfamily. The large effect QTNs Chr7AS: AX-95249973 and Chr7BS: AX-95123855 associated with FOLRN were involved in plant growth, development and adaptation (Supplementary Table S6).

## 3. Discussion

Root traits play a major role in resource uptake, particularly with respect to water access and uptake in the context of drought tolerance. In this way, root traits help maintain crop yield under limited water environments. To realize maximum yield in wheat, a well-developed root system is warranted [28,35]. The discovery of a novel source of germplasm and new alleles to improve RSA and introgression of new traits into adapted but otherwise susceptible phenotypes are a desirable approach toward breeding for drought tolerance. Different strategies were adopted for early screening of the wheat RSA, assuming that genotypes with a diverse nature of root architecture at the seedling stage would also respond in a similar way at the adult stage when water and/or nutrients become scarce for grain yield [36]. We studied the root and shoot behaviors at the seedling stage under controlled conditions in perlite vermiculite mixtures in pots. This method of screening was considered a reliable method to examine the root system variations compared to field conditions, where there are several confounding impacts and extracting and measuring roots is difficult. Genetic variability in root systems during seed germination and seedling development is a key trait for seedling establishment and early vigor under water stress and resource poor environments. Improved root traits during this key stage will impact crop emergence and seedling establishment resulting in a good plant population which is one of the key components of yield under both optimal conditions and stress conditions.

### 3.1. Phenotypic Variability

The significant variation observed for various RSA features and shoot traits could be attributed to diversity among the genotypes of the association panel due to their diverse genetic background and wide geographical distributions. The association panel genotypes were collected from various parts of India and around the world. Further, a broad range of variation was detected for individual RSA traits. The highest and lowest morphometric values for various RSA traits (Table 1) reflected the level of diversity within the gene pool and could be promising material to improve root traits through breeding. High coefficient of variation (%) for FOLRN, SOLRN, LRS, LRD, RV, RDW and SRL (>30%) revealed wide variability of these traits in the AM panel. The broad sense heritability (*H*^2^) of the trait is a critical parameter determining their utility in breeding. We observed high heritability for traits such as SOLRN, SDW, RDW, SL, RL, AD, RV and moderate heritability for traits such as, LRS, RLD, SRN, RSDWR, SRL, TRS (Supplementary Table S2). Li et al. [37] reported moderate to high *H*^2^ for root and shoot traits, ranging from 56.0% to 94.6%.

For acquisition of water and nutrients from the deep zone of soil, RV with a greater number of root tips is essentially required [8,10]. Further, it would be desirable to have genotypes with longer RV, RDW, and other associated parameters so they could extract water and maintain growth under moisture stress environments. The genotype IC406521 from Uttarakhand (India) showed the highest mean value of RV (0.35 cm^3^), and lowest AD (0.31 mm), thus its root distribution occupied a larger area of soil. Further, the genotype IC539574 ranked one with the value of 240.66 for FOLRN whereas the genotype IC82425A recorded the highest mean value of 490.33 for SOLRN. Similarly, top ranking genotypes for RLD, LRS, LRD and AD were IC542076 (0.75 cm^−2^), IC82425A (0.86), IC29008 (5.89 cm^−1^) and EC187159 (0.49 mm), respectively. These genotypes could serve as donors of these traits for crop improvement to combat drought stress. The genotypes with the highest AD are considered best for drought stress tolerance due to large xylem vessels which can extract more water and nutrients from deep soil layers [12,38]. We recorded variation for SRN in our diverse panel, ranging from 2 to 6 which was also observed by Djanaguiraman et al. [14]. However, Cane et al. [36] reported 4 to 6 seminal roots in elite wheat varieties which might be due to less variability. We identified donors for multiple traits. For example, the genotype EC426644, an Australian cultivar (Tincurrin), showed high performance for traits, namely TRS, RLD, FOLRN and RSDWR. Bustos et al. [39] also reported Tincurrin cultivar with high root system size in their study.

### 3.2. Multivariate Analyses

Correlations between root and shoot traits shows the balance between the organs of roots and shoots and resource partitioning between the above ground and below ground plant parts [40,41]. In this case, FOLRN and SOLRN exhibited strong correlations with all the traits except AD and SRL. FOLRN directly or indirectly contributes to the variability in root morphological traits which increase water use efficiency at critical stages of plant growth through more absorption of water and nutrients from soil sub layers. The inverse relationship between AD with other traits observed in the present study was in line with the previous studies on flax (*Linum usitatissimum* L.) and *Arabidopsis thaliana* [42,43]. This is because fine roots are the main root feature, and the number of root tips increases the root surface area and volume, hence enhancing the absorption efficiency of water and nutrients [8,10]. A high correlation between RV and RDW is obvious. Moreover, a high correlation between shoot and root dry weights observed might be due to supply of nutrients from root to the shoot parts, as is evident in the case of rice [44]. One of the easily scorable key traits, SRN, showed a high correlation with a majority of the root traits, suggesting its utility to provide a broad idea about the root system. The high correlation between root and shoot traits is due to reliance of crops on the root system for water and nutrient uptake.

Based on PCA, RV, RDW, SOLRN, RLD, TRS, RL, LRS, FOLRN, SRN and RSDWR were major contributors to PC1 and most significantly associated with genotypic variations. Likewise, SDW and SL are major contributors to PC2. Hence these traits need to be focused on root studies. RV indicates the proliferation of roots which is essential for plants to uptake more water and nutrients from the soil, whereas linear root elongation may be quite useful in extracting water from deeper soil layers in the event of drought. However, RDW varies among genotypes with varying RL. Therefore, it would be desirable to have genotypes with high RL as well as RDW for a more efficient root system. Higher root density is found to be useful for the plants to uptake more nutrients and therefore the plants with a deeper root system would be more advantageous for drought tolerant genotypes [45,46]. Clustering of genotypes in six groups was also evident in a two-dimensional PCA biplot based on root and shoot morphological traits. Genotypes from two contrasting clusters 1 and 6 could be used in crossing program for genetics studies, mapping population development and trait introgression.

### 3.3. Genome Wide Association and Candidate Genes Identification

Identification of a novel source of genes and genomic regions associated with RSA traits at the seedling stage is expected to boost development of high yielding drought tolerant varieties. In this context, historical wheat germplasm collections maintained in gene banks could prove valuable genetic resource for searching genes/genomic regions using association genetics approach. In this study, we used a diverse panel of 136 wheat genotypes including landraces, varieties, local collection, synthetic germplasm and elite breeding line for mapping RSA and shoot traits. For conducting GWAS, ML-GWAS models were used as these are considered superior to SL-GWAS models for mapping complex traits due to the fact that in ML-GWAS models, all-marker effects are simultaneously estimated. Moreover, unlike SL-GWAS models, these do not require testing of identified associations using stringent multiple testing corrections that generally result in rejection of significant associations [47]. Using six different ML-GWAS models, 456 QTNs were detected for 15 RSA features and shoot traits, distributed over 21 chromosomes. The phenotypic variation estimated (PVE) (r^2^) for the traits ranged from 0.12% to 38.67%, indicating that RSA traits are controlled by multiple loci with small to moderate effects. This also revealed the complex genetic control of these traits at an early stage of crop growth. Among the six ML-GWAS models used in our analysis, the pLARmEB model was the most powerful, which revealed the maximum number of associations (194) whereas the FASTmrEMMA was the least powerful as it detected the lowest number of MTAs (18). These findings were consistent with the observation of the Safdar et al. [48] who used ML-GWAS models to dissect agronomic traits in bread wheat.

Among the root traits, TRS was found to be associated with drought tolerance in wheat due to spreading of roots in the soil and its effect on the resource uptake [49]. The SNP marker AX-94952472, AX-95105488 and AX-95119337 on chr2AS, 4AL and 7BS were associated with TRS as well as RLD with phenotypic variance in the range of 3.29–11.23%. Root length is an important parameter that determines the ability of a plant to capture water from deeper soil layer [50]. Eight QTNs for RL were detected with more than two models, out of which *Q.RL-6BL* was consistently detected in five models. Further, this marker was pleiotropic and was associated with RV and RLD which were positively associated traits. Two reliable QTNs for RL (*Q.RL-6BL* and *Q.RL-7AL*) were not documented in earlier studies. There, they are considered novel QTNs, whereas other QTNs were reported to be associated with wheat seedling root development under abiotic stresses and phosphorus starvation [51,52]. For traits, FOLRN and SOLRN, two and nine QTNs were identified, respectively. Lateral roots play an important role for foraging water from a shallow depth. Wheat cultivation in the dry area requires more lateral roots at deeper layers for absorption of nutrients and water from deep soil layers. Our analysis revealed a pleiotropic marker, AX-95123855 on 7BS associated with RSA traits FOLRN, SOLRN and LRS. This marker was located within a gene (*TraesCS7B02G086500)* that encodes Calcineurin which was reported to control inward ion flux in the root, a process essential for plant survival and growth [53].

The SRL is a widely used morphological parameter for root trait which characterizes economic aspects of root system, demonstrating the role of root mass for nutrient acquisition. Genotypes with large SRL have thin roots that increase the surface area per unit root volume enhancing water and nutrient uptake efficiencies [54]. Furthermore, genotypes with small root diameter and large RLD at depth are better adapted to drought conditions [55]. Our study identified 2 QTNs for SRL, one each on 5AL and 2BL. Of these, QTN on 2BL (*Q.SRL-2BL*) was associated with a gene encoding for Formin-like protein, a family of actin organizing protein involved in root hair development [33]. Further, RLD is considered a primary driver of drought avoidance and enables complete uptake of the soil moisture. Breeding for plants with increased RLD in medium and deep layers and less RLD in shallow soil layers have been proposed as an efficient growth strategy where deep water could be available to crops during late maturity [45,56,57,58]. Nine QTNs were identified for RLD in our study. Root diameter is another critical parameter determining nutrient uptake and transport. In this study, we identified as many as eleven consistent QTNs for AD and all of them had minor effects, suggesting complex genetic regulation of this trait. Among these, two QTNs *Q.AD-1BL* (AX-94620468) and *Q.AD-6AL* (AX-95244609) were detected in three or more models and could represent important genomic regions controlling this trait. Moreover, we found that marker AX-95244609 for QTN *Q.AD-6AL* was associated with other traits such as SOLRN and LRS, and thus could be a potential target for improvement of more than one RSA traits.

Another important trait is SRN, which emerges first from coleorhiza of seed embryo. The gravitropic response of wheat seminal roots was heritable and suggested to be under control of a single dominant gene [59]. Soriano et al. [60] reported meta-QTNs for SRN in wheat on 2A, 2B, 3B, 4A, 5A, 7A and 7B. In this study, a total of six novel QTNs for SRN were identified with the QTNs on 2D (*Q.SRN-2DL*: AX-94551988) having consistency in four methods. We found that SNP marker AX-94551988 associated with *Q.SRN-2DL* was located within gene *TraesCS2D02G478700* encoding for histone-lysine N-methyltransferase. In a previous study on *Arabidopsis,* histone-lysine N-methyltransferase gene was reported to inhibit lateral root development [61]. Based on this, it could be suggested that identified Histone-lysine N-methyltransferase candidate gene might participate in regulating SRN through modification of histone proteins.

Moreover, root biomass accumulation at the seedling stage is beneficial for drought escape as it could enable efficient uptake of water and nutrient from soil. In previous studies, RDW QTLs have been identified under both normal and drought stress conditions [60]. Among the four QTNs for RDW identified in this study, the one on 7AS (*Q.RDW-7AS*) explained maximum percentage of phenotypic variance (18.35–18.60%) and coincided with a meta-QTL, *root_MQTL_68* [60], suggesting this is an important genomic region associated with root trait and its role can be further validated. Among the studied traits, RSDWR is considered the most important as it indicates ability of a genotype to absorb water and nutrients from the soil. Generally, plants have higher root shoot ratio under nutrient deficit soils, suggesting more allocation of resources for the development of root so the plant can extract nutrients from deep and wider zone [62]. Studies have identified genomic regions for the RSDWR trait on various chromosomes of wheat under both control as well as nutrient deficit conditions [60]. Our analysis revealed three QTNs for RSDWR, one each on 2A, 2B and 3B respectively, however each of them have small effect, indicating limited potential for their utilization in breeding programs.

The underground root traits significantly affect development of above ground traits including shoot traits such as, SDW and SL, and thus grain yield. We could identify six and five reliable QTNs for SDW and SL, respectively. The strongest QTN for SDW was identified on 4A, *Q.SDW-4AL* which explained phenotypic variance in the range of 8.09–19.05%, and was found reliable, as detected by using all five models. The SNP marker associated with this QTN was located within a gene *TraesCS4B02G095100* that encodes for an F-box-like domain superfamily. F-box family proteins play a diverse role in plant growth and development and could be critical for shoot growth as their expression has also been detected in the shoot tissues of plant species [49].

Applications of root phenotype-genotype association through GWAS has enabled the identification of important QTNs for root traits that impact shoot traits including yield [63,64]. Markers significantly associated with these traits can be used in marker assisted backcross breeding for varietal improvement. Pleiotropic loci with consistent effects should be amenable to MAS for many traits together. Further, our study provides understanding into phenotype–genotype associations for early root and shoot traits of diverse wheat genotypes by identifying QTNs and proposing plausible candidate genes for future investigations.

## 4. Materials and Methods

### 4.1. Experimental Material and Design

**Figure 6:**
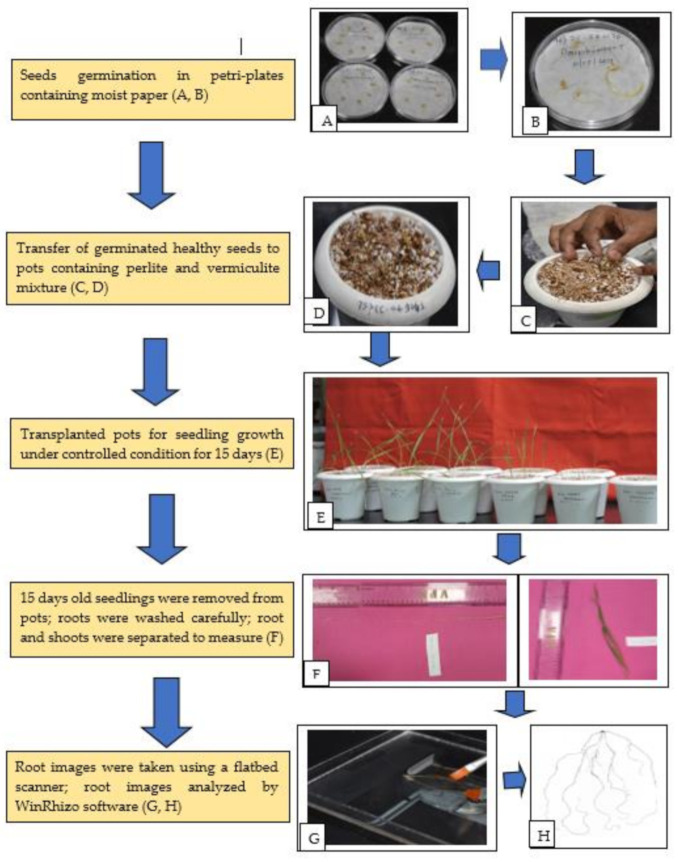
Flow diagram of phenotyping protocol. (**A**) Sterilized wheat seeds for germination; (**B**) germinated seeds after two days; (**C**) transferring healthy seedlings to pot containing perlite and vermiculite; (**D**) transplanted seedlings; (**E**) different stages of seedlings growth under controlled condition; (**F**) manual measurement of root and shoot length; (**G**) root images captured by using flatbed scanner; (**H**) scanned images of roots. The experimental design adopted for screening was a completely randomized design (CRD) with three replications including two checks (C306 and HD2967). For each replication five biological replicates were used in each pot. The experiment was done in batches for 20 genotypes at each time. Before germination, 20 seeds of each accession were washed carefully and thoroughly with double distilled water and surface sterilized with 0.5% Sodium hypochlorite solution for 30 s. The sterilized seeds were then washed with double distilled water three to four times to remove any trace of adhering chemicals. The seeds were placed well spread in a thoroughly moist germination paper/filter paper taken in a petri dish and allowed to germinate under a growth chamber at 22 ± 1 °C room temperature in the dark.

Phenotyping of 140 wheat germplasm of diverse panel including mini core subset was carried out at the Indian Council of Agricultural Research—National Institute of Plant Biotechnology (NIPB), New Delhi (India) as pot screening in growth chambers under controlled environmental conditions during Kharif season (June–September), 2019. Detailed information about the origin and pedigree of the material is given in Supplementary Table S1. Root morphology and root system architecture (RSA) along with shoot traits were studied by destructive methods at early seedling stage (Fifteen-day-old seedlings). Figure 6 describes the steps and methodology used in this experiment.

In this experiment, five germinated wheat seeds were sown initially in pots (4-inch diameter) containing perlite and vermiculite (1:2 ratio *v*/*v*). Germinated seeds that were two days old with primary roots of 1 cm in length were placed inside the pot with the root radical facing down at equal depth. The Murashige–skoog (MS) liquid media were supplemented with 8 mM NO_3_^−^ as nutrient media at uniform intervals during the entire growth period. The calcium concentration in all media was kept constant by adjusting the amount of CaNO_3_. Murashige–skoog (MS) N^+^ nutrient solution was prepared as per the given composition and stored in 4 °C. For one liter of working solution preparation, 100 mL of MS media, 4 mL of CaNO_3_ and the remaining 896 mL of distilled water constituted 1 L of solution, which were added in equal proportion to all the genotypes grown in controlled conditions at equal intervals. In the growth chamber, 150–200 μmol photon/m^2^/s light intensity, 10/14 dark/light hours, 70% relative humidity (RH) and at 22 ± 1 °C conditions were maintained as described by Sinha et al. [65].

### 4.2. Root and Shoot Traits Measured for Phenotyping

After completing 15 days of growth in the controlled growth conditions, the seedlings were harvested from the pots by removing the perlite + vermiculite mixture and plants were separated carefully without damaging the roots and shoots. The plants were immediately placed on a tray to wash out the adhering perlite + vermiculite particles by running tap water with the utmost care so that to avoid breakage of any roots. The entire seedling was carefully spread on blotting paper and maximum root length and shoot length were measured with the help of a length measuring scale. The root was then extracted intact by cutting at the collar region using a sharp blade and the roots were placed in a tray containing distilled water. The roots were then individually scanned in an Epson Perfection V 700 Photo^®^ flatbed scanner at a resolution of 400 dpi modified for this purpose (Regent Instruments Inc., Quebec, QC, Canada) as per the manufacturer’s guidelines. The root images from the scanner were analyzed with customized software WinRHIZO™ (Regent Instruments Inc., Quebec, QC, Canada) [66]. Various root trait data were recorded by software which were later transformed in major RSA traits measured manually using published protocols [32,67,68,69] (Table 1).

### 4.3. Statistical Analyses of Phenotypic Data

Mean data across the replications were used as the input data for statistical analyses and GWAS analysis. Descriptive statistics and frequency distribution were analyzed to check range of variability among the traits. Pearson’s correlation coefficient, cluster analysis and PCA was performed using SAS software version 9.3 (JMP) program (SAS Institute, Cary, NC, USA). Heritability (H^2^) was estimated from the analysis of variance.

### 4.4. DNA Extraction and SNP Genotyping

Genomic DNA was extracted from 15-day-old wheat seedlings using CTAB methods [70]. DNA quality was checked using Nanodrop^TM^ 2000 (Thermo Fisher Scientific, Wilmington, DE, USA). Samples with good quality DNA were genotyped using Axiom^®^ Wheat Breeders’ Array (Thermo Fisher Scientific, Wilmington, DE, USA) according to the procedure described by Affymetrix (Axiom^®^ 2.0 Assay for 384 samples P/N 703,154 Rev. 2). SNP markers with >10% missing data and <10% MAF were excluded. Identified SNPs were localized on a wheat genome assembly International Wheat Genome Sequencing Consortium (IWGSC) RefSeq version 1.0 using BLASTn program with default parameters.

### 4.5. Population Structure and LD

Population structure was estimated with 525 unlinked SNP markers nearly uniformly distributed across the wheat genome using the Bayesian model-based approach implemented in STRUCTURE program version 2.2 (Pritchard Lab, Stanford University, Stanford, CA, USA) [71]. A burn-in of 20,000 iterations followed by 50,000 Monte Carlo Markov Chain (MCMC) was run to estimate the number of subpopulation (k) in a putative range of k = 1 to 10. The subpopulation number was estimated using an ad hoc statistic delta k based on the rate of change in log probability of data between successive values [72]. The squared allele frequency correlation (r^2^) between SNP markers was used to estimate LD across sub-genome A, B, and D using TASSEL v5.0 (Buckler’s Lab, Ithaca, NY, USA) [73]. LD decay across the three sub-genomes and whole genome level was estimated as the physical distance between SNPs where average r^2^ reduced to half of the maximum LD value.

### 4.6. Genome Wide Association Analysis

The use of multi-locus methods that capture small effect loci in complex polygenic traits such as in plant roots and shoots have recently become a popular and feasible approach. To benefit the algorithmic merits of different models and support results of one by another, it is also advantageous to apply multiple methods [49]. GWAS analysis was performed using six multi locus GWAS methods within mrMLM [74], FASTmrMLM [75], FASTmrEMMA [76], pKWmEB [77], pLARmEB [78] and ISIS EMBLASSO [79], which were included in the R package mrMLM v3.1 [80]. All parameters were set at default values in this GWAS. The critical thresholds of significant association for the six methods were set as LOD score 3.00 or >3.00. The most significant QTNs, detected in at least two methods, were considered as reliable QTNs. The associated SNPs and their putative underlying genes were illustrated on the wheat chromosomes using Map Chart 2.3, (https://www.wur.nl/en/show/Mapchart.htm accessed on 8 June 2021) [81]. The favorability of alleles at QTNs detected by at least four of the multi-locus models was illustrated using a box plot based on mean phenotypic value of genotypes with each allele.

### 4.7. Identification of Potential Candidate Genes

SNPs (probe sequences) that were significantly associated with root architecture traits were searched against the *Triticum aestivum* genome assembly IWGSC-refseq version1.0 in online web resource Ensemble plants (https://plants.ensembl.org/Triticum_aestivum/Tools/Blast, accessed on 14 November 2020) using BLASTn with default parameters to identify potential candidate genes. BLAST2GO tool was used to get annotation of expressed transcripts [82].

## 5. Conclusions

This study revealed wide variability for RSA and shoot traits at the seedling stage in the studied association panel. Ten top and bottom performing lines for 15 traits were identified for use in genetics, development of mapping population and introgression studies. Based on PCA, several traits such as RV, RDW, SOLRN, RLD, TRS, RL, LRS, FOLRN, SRN and RSDWR, were the most influential traits for phenotypic variations. The GWAS analysis enabled genetic detection of RSA traits and revealed 11 pleotropic loci associated with correlated traits. Further, putative candidate genes were identified from the associated genomic region that could be validated using a functional genomics approach. Development of wheat cultivars possessing superior root traits will play an important role in enhancing drought tolerance under water stress conditions. The large variability found in Indian germplasm for RSA traits and the novel genomic regions regulating them makes this germplasm a valuable source for improving root architecture, which plays a significant role in absorption and uptake of water and nutrients and increases crop productivity.

## Figures and Tables

**Table 1:** Root system architecture and shoot traits along with their basic statistics of the wheat accessions in the diverse association panel.

S. No.	Trait Name	Abbreviation	Trait Description	Unit	Range	Mean ± SE	CV (%)
1	Total root size	TRS	Sum of the path length of seminal including primary roots and lateral roots (LRs)	cm	65.10–378.63	242.94 ± 5.35	26.06
2	First order lateral root number	FOLRN	Number of first order LRs (emerging from primary and seminal roots)	-	14.33–240.67	95.22 ± 3.58	44.50
3	Second order lateral root number	SOLRN	Number of second order LRs (emerging from first-order LRs)	-	6.67–490.33	165.57 ± 8.97	64.10
4	Lateral root size	LRS	First order length + second order length divided by total root size (TRS)	-	0.02–0.87	0.39 ± 0.01	38.39
5	Lateral root density	LRD	First-order LR no. divided by PRP	cm^−1^	0.81–5.89	2.81 ± 0.08	35.67
6	Root length density	RLD	TRS divided by volume of pot (500 cm^3^)	cm^−2^	0.11–0.76	0.49 ± 0.01	26.30
7	Average diameter	AD	Projected area divided by TRS	mm	0.31–0.49	0.38 ± 0.01	8.46
8	Root volume	RV	π × (Half of the avg. diameter/2) 2 × TRS	cm^3^	0.02–0.35	0.16 ± 0.01	37.62
9	Seminal root number	SRN	Primary root bursts through the coleorhiza	-	2.67–6.00	5.06 ± 0.04	10.29
10	Shoot dry weight	SDW	Shoot dry weight	mg	5.02–60.41	35.58 ± 0.88	29.18
11	Root dry weight	RDW	Root dry weight	mg	1.07–23.06	10.76 ± 0.32	35.14
12	Shoot length	SL	Shoot length	cm	13.57–40.77	30.61 ± 0.41	15.97
13	Root length	RL	Root length	cm	9.67–37.50	24.81 ± 0.54	25.83
14	Root shoot dry weight ratio	RSDWR	Ratio of root to shoot dry weight	-	0.16–0.59	0.31 ± 0.01	26.07
15	Specific root length	SRL	Total root length per unit root dry mass	cm mg^−1^	1.32–9.10	2.52 ± 0.07	34.02

**Table 2:** Distribution of 16,616 SNPs and identified linkage disequilibrium (LD) on 21 chromosomes in 136 wheat genotypes.

Chromosome	Size (Mb)	No. of SNP	Average Number of SNPs per (Mb)	Average LD (r^2^)	No. of Marker Pairs in Perfect LD (r^2^ = 1)
1A	594.1	911	1.53	0.206	2134
1B	689.85	987	1.43	0.268	3511
1D	495.45	895	1.80	0.298	3923
2A	780.8	952	1.22	0.241	999
2B	801.26	1178	1.47	0.159	819
2D	651.85	1037	1.59	0.226	1813
3A	750.84	663	0.88	0.114	274
3B	830.83	1003	1.20	0.149	368
3D	615.55	579	0.94	0.098	121
4A	744.59	573	0.76	0.122	310
4B	673.62	447	0.66	0.111	197
4D	509.86	287	0.56	0.087	73
5A	709.77	737	1.03	0.122	368
5B	713.15	1125	1.57	0.189	1769
5D	566.08	769	1.35	0.147	713
6A	618.08	657	1.06	0.157	655
6B	720.99	846	1.17	0.147	354
6D	473.59	622	1.31	0.112	255
7A	736.71	862	1.17	0.122	401
7B	750.62	757	1.00	0.126	269
7D	638.69	729	1.14	0.099	202

**Table 3:** Common quantitative trait nucleotides (QTNs) of root and shoot traits identified using different multi-locus genome-wide association studies (GWAS) methods.

S. No	QTN	Trait	Marker	Allele	CHR	Physical Position (bp)	LOD Score	R^2^ (%)	Method
1	*Q.TRS-2AS*	TRS	AX-94952472	G/C	2AS	8181794	3.29–3.75	9.77–17.72	1,5
2	*Q.TRS-4AL*	TRS	AX-95105488	A/C	4AL	631903354	3.38–5.01	8.82–10.85	2,4
3	*Q.TRS-7BS*	TRS	AX-95119337	G/A	7BS	138882791	6.81–10.73	17.10–22.22	2,4
4	*Q.FOLRN-7AS*	FOLRN	AX-95249973	G/A	7AS	54997993	3.10–4.92	5.22–6.83	1,2
5	*Q.FOLRN-7BS*	FOLRN	AX-95123855	T/C	7BS	99635031	3.59–4.41	6.12–8.62	4,5
6	*Q.SOLRN-1AL*	SOLRN	AX-95102105	A/G	1AL	474573230	3.24–4.58	1.68–3.65	1,2,4
7	*Q.SOLRN-1BL*	SOLRN	AX-95077960	G/A	1BL	572301473	3.55–8.97	2–4.15	1,2,4,5
8	*Q.SOLRN-1DS*	SOLRN	AX-94448890	T/C	1DS	10741698	4.44–6.92	2.47–4.12	1,2,4,5
9	*Q.SOLRN-3BL*	SOLRN	AX-94516395	C/T	3BL	738699225	4.34–5.58	2.61–3.99	1,2,4
10	*Q.SOLRN-3BL*	SOLRN	AX-95150666	G/C	3BL	664019441	10.31–14.84	27.09–38.60	1,2,4,5
11	*Q.SOLRN-3DL*	SOLRN	AX-94450588	A/G	3DL	367309893	8.17–10.84	21.14–26.16	4,5
12	*Q.SOLRN-6AL*	SOLRN	AX-95244609	T/C	6AL	599035159	4.61–6.78	1.88–3.29	1,2,4,5
13	*Q.SOLRN-7BL*	SOLRN	AX-94564853	A/G	7BL	673962683	4.30–5.98	9.92–18.50	1,2,4
14	*Q.SOLRN-7BS*	SOLRN	AX-95123855	T/C	7BS	99635031	4.49–4.69	1.60–2.17	4,5
15	*Q.LRS-2DS*	LRS	AX-94471646	A/G	2DS	13929712	3.31–7.30	1–2.40	2,4
16	*Q.LRS-3AS*	LRS	AX-94961347	C/T	3AS	184635201	7.15–15.48	7.47–12.41	1,4,5
17	*Q.LRS-3BL*	LRS	AX-94480990	G/T	3BL	417261367	6.63–15.77	1–6.15	2,4
18	*Q.LRD-5BS*	LRS	AX-94706358	A/C	5BS	69134215	3.41–13.25	3.47–8.20	1,4,5
19	*Q.LRS-6AL*	LRS	AX-95244609	T/C	6AL	599035159	3.27–4.21	0.12–0.2	4,5
20	*Q.LRD-7BL*	LRS	AX-94528392	G/A	7BL	675314495	4.92–9.18	16.43–30.67	1,4,5
21	*Q.LRD-7BS*	LRS	AX-95123855	T/C	7BS	99635031	3.35–17.24	4.17–8.35	1,2,4,5
22	*Q.LRD-4AL*	LRD	AX-94841365	G/A	4AL	725751499	4.90–5.56	12.04–13.40	4,5
23	*Q.LRD-5DL*	LRD	AX-94571501	T/C	5DL	472635782	3.46–5.69	5.74–11.60	3,4,5
24	*Q.LRD-7BL*	LRD	AX-94763902	T/C	7BL	552120841	3.58–6.43	6.83–10.02	2,4,5
25	*Q.RLD-1BL*	RLD	AX-94728516	T/C	1BL	491241476	6.01–28.19	0.45–4.15	4,5
26	*Q.RLD-2AS*	RLD	AX-94952472	G/C	2AS	8181794	3.67–9.52	0.98–4.23	4,5
27	*Q.RLD-3BS*	RLD	AX-94516395	C/T	3BL	738699225	3.36–3.46	1.66–1.93	4,5
28	*Q.RLD-3DL*	RLD	AX-94544285	C/G	3DL	594478810	5.88–21.54	3.94–6.33	2,4
29	*Q.RLD-4AL*	RLD	AX-95105488	A/C	4AL	631903354	3.26–8.06	5.97–15.44	1,4
30	*Q.RLD-5AL*	RLD	AX-95659861	G/A	5AL	565754230	3.49–6.68	1.89–2.57	2,5
31	*Q.RLD-5DS*	RLD	AX-95160709	G/T	5DS	10471964	3.49–13.73	0.16–4.38	4,5
32	*Q.RLD-6BL*	RLD	AX-94502864	G/A	6BL	710324542	9.73–14.54	1.14–8.22	4,5
33	*Q.RLD-7BS*	RLD	AX-95119337	G/A	7BS	138882791	6.71–11.23	12.20–17.08	1,4,5
34	*Q.AD-1AL*	AD	AX-94932678	A/G	1AL	557954056	3.67–6.82	2.42–5.92	4,5
35	*Q.AD-1BL*	AD	AX-94620468	A/G	1BL	586302698	5.16–8.86	3.31–7.79	2,4,5
36	*Q.AD-1BS*	AD	AX-94668789	G/A	1BS	96515530	3.67–5.97	0.17–1.53	2,5
37	*Q.AD-2DL*	AD	AX-94647816	A/C	2DL	641980126	4.58–22.75	3.53–7.08	2,4
38	*Q.AD-3AS*	AD	AX-94996868	C/T	3AS	1030506	6.25–16.45	1.67–8.54	2,4
39	*Q.AD-3BS*	AD	AX-94886515	T/C	3BS	129437930	3.90–9.27	1.55–2.72	2,5
40	*Q.AD-3DS*	AD	AX-94726849	C/T	3DS	81391081	6.70–7.87	0.21–2.18	2,4
41	*Q.AD-4AL*	AD	AX-94740863	T/C	4AL	725802675	3.10–5.14	0.12–3.54	4,5
42	*Q.AD-6AL*	AD	AX-95244609	T/C	6AL	599035159	3.38–10.62	3.03–5.88	2,3,4,5
43	*Q.AD-6BS*	AD	AX-94691127	G/A	6BS	288419550	4.97–5.80	0.18–1.28	4,5
44	*Q.AD-6DL*	AD	AX-94853162	G/A	6DL	470294593	3.70–4.51	0.39–1.65	4,5
45	*Q.RV-2BS*	RV	AX-95227366	G	2BS	46089220	3.25–1152.63	11.40–13.56	1,2,4
46	*Q.RV-4AL*	RV	AX-95105488	A/C	4AL	631903354	3.97–1166.09	2.06–8.08	1,2,3
47	*Q.RV-4BS*	RV	AX-94648074	C/T	4BS	140929724	3.45–1122.58	2.72–4.81	1,2,4
48	*Q.RV-5BL*	RV	AX-94439232	C/A	5BL	287624152	3.80–1152.24	1.47–5.31	1,2,4,5
49	*Q.RV-6AL*	RV	AX-95162623	C/T	6AL	611851405	3.76–7.56	0.18–2.61	4,5
50	*Q.RV-6AS*	RV	AX-94687596	A/G	6AS	959041	5.10–7.87	0.97–2.20	4,5
51	*Q.RV-6AS*	RV	AX-94893701	A/G	6AS	24857477	5.21–9.45	0.70–1.23	4,5
52	*Q.RV-6BL*	RV	AX-94502864	G/A	6BL	710324542	4.54–1136.81	0.52–5.93	2,4,5
53	*Q.RV-7AL*	RV	AX-94664277	C/T	7AL	689920746	4.61–1155.16	5.53–11.20	1,2,4,5
54	*Q.SRN-2DL*	SRN	AX-94551988	A/G	2DL	579965138	4.27–7.94	6.83–17.74	1,3,4,5
55	*Q.SRN-3BS*	SRN	AX-94486290	T/C	3BL	477885835	5.68–6.68	5.82–6.51	1,2
56	*Q.SRN-3DL*	SRN	AX-94382595	C/T	3DL	392956661	3.19–4.40	0.31–8.32	2,4
57	*Q.SRN-4DL*	SRN	AX-95157799	A/G	4DL	461218017	5.66–7.19	17.38–20.91	1,2
58	*Q.SRN-6AL*	SRN	AX-94637211	A/G	6AL	611576606	3.08–4.87	11.77–14.95	1,2
59	*Q.SRN-6AL*	SRN	AX-94790960	C/A	6AL	600131055	6.19–9.13	23.20–28.08	1,2
60	*Q.SDW-3DS*	SDW	AX-94439998	T/C	3DS	85060915	4.29–4.29	8.15–9.74	1,2
61	*Q.SDW-4AL*	SDW	AX-94470023	T/C	4AL	581216490	4.49–6.61	8.09–19.05	1,2,3,4,5
62	*Q.SDW-4BS*	SDW	AX-95630040	A/G	4BS	12893614	3.25–8.40	5.43–11.57	3,4,5
63	*Q.SDW-5BL*	SDW	AX-94484139	T/C	5BL	289567014	4.72–4.87	5.64–5.92	4,5
64	*Q.SDW-7AL*	SDW	AX-94664277	C/T	7AL	689920746	3.36–4.03	4.39–5.30	4,5
65	*Q.SDW-7BL*	SDW	AX-95165787	C/A	7BL	591053449	3.11–3.13	6.54–7.06	4,5
66	*Q.RDW-1BL*	RDW	AX-94925225	T/C	1BL	372996241	4.49–4.97	4.87–7.59	4,5
67	*Q.RDW-5AL*	RDW	AX-94501549	C/T	5AL	672339596	4.29–4.91	4.71–7.45	2,3,4
68	*Q.RDW-6AS*	RDW	AX-94809955	C/T	6AS	631698	4.67–5.39	4.37–5.67	2,4
69	*Q.RDW-7AS*	RDW	AX-94386260	G/A	7AS	269093921	3.65–4.35	18.35–18.60	1,2
70	*Q.SL-1AL*	SL	AX-94929421	T/C	1AL	550331092	5.63–5.66	5.16–5.39	1,2
71	*Q.SL-2AL*	SL	AX-95076063	T/C	2AL	555820355	3.52–4.91	3.68–8.24	1,3,5
72	*Q.SL-4BS*	SL	AX-95012217	C/T	4BS	97922535	4.71–6.36	5.06–5.74	1,5
73	*Q.SL-5BL*	SL	AX-95092434	C/G	5BL	579153579	4.16–5.20	5.57–8.61	1,2
74	*Q.SL-7BS*	SL	AX-94876335	G/A	7BS	144484919	4.04–5.48	7.23–8.27	1,2
75	*Q.RL-1DS*	RL	AX-94448890	T/C	1DS	10741698	4.02–5.52	3.67–7.89	1,2,5
76	*Q.RL-2BS*	RL	AX-95227366	G	2BS	46089220	3.68–5.77	8.59–15.76	1,4
77	*Q.RL-6BL*	RL	AX-94502864	G/A	6BL	710324542	4.63–6.06	4.68–9.41	1,2,3,4,5
78	*Q.RL-6BS*	RL	AX-94539094	A/C	6BS	8387528	3.52–6.77	2.83–17.15	1,2,5
79	*Q.RL-6BS*	RL	AX-94900754	A/G	6BS	157792843	3.27–5.44	5.26–9.16	1,3
80	*Q.RL-7AL*	RL	AX-95241843	G/T	7AL	610498024	6.31–6.50	5.55–5.97	1,2
81	*Q.RL-7BL*	RL	AX-94528392	G/A	7BL	675314495	3.21–8.52	15.42–30.83	1,5
82	*Q.RL-7DL*	RL	AX-94861078	A/G	7DL	614276051	3.36–5.01	3.12–4.24	3,4
83	*Q.RSDWR-2AL*	RSDWR	AX-94430108	C/T	2AL	509357786	5.19–14.68	0.55–1.38	2,4
84	*Q.RSDWR-2BL*	RSDWR	AX-94856412	C/T	2BL	754864363	4.85–103.96	0.58–1.95	4,5
85	*Q.RSDWR-3BS*	RSDWR	AX-94691217	A/G	3BS	8029967	8.14–83.91	0.02–3.22	2,4
86	*Q.SRL-5AL*	SRL	AX-94748697	A/G	5AL	406534846	3.37–6.01	4.81–8.10	2,4,5
87	*Q.SRL-2BL*	SRL	AX-94457792	T/C	2BL	576083471	8.12–19.40	64.33–31.92	1,2,4,5

TRS, total root size (cm); FOLRN, first order lateral root number; SOLRN, second order lateral root number; LRS, lateral root size; LRD, lateral root density (cm^−1^); RLD, root length density cm^−2^; AD, average diameter (mm); RV, root volume (cm^3^); SRN, seminal root number; SDW, shoot dry weight (mg); RDW, root dry weight (mg); SL, shoot length (cm); RL, root length (cm); RSDWR, root shoot dry weight ratio; SRL, specific root length (cm mg^−1^). ML-GWAS methods (MrMLM-1, FASTMrMLM-2, FASTMrEMMA-3, pLARmEB-4, ISIS EM-BLASSO-5).

**Table 4:** Pleiotropic quantitative trait nucleotides (QTNs).

S. No.	Marker	Traits	Chromosome	Position (Mb)
1	AX-94448890	SOLRN, RL	1DS	10.7417
2	AX-94502864	RLD, RV, RL	6BL	710.3245
3	AX-94516395	SOLRN, RLD	3BL	738.6992
4	AX-94528392	LRS, RL	7BL	675.3145
5	AX-94664277	RV, SDW	7AL	689.9207
6	AX-94952472	TRS, RLD	2AS	8.181794
7	AX-95105488	TRS, RLD, RV	4AL	631.9034
8	AX-95119337	TRS, RLD	7BS	138.8828
9	AX-95123855	FOLRN, SOLRN, LRS	7BS	99.63503
10	AX-95227366	RV, RL	2BS	46.08922
11	AX-95244609	SOLRN, LRS, AD	6AL	599.0352

TRS, total root size (cm); FOLRN, first order lateral root number; SOLRN, second order lateral root number; LRS, lateral root size; RLD, root length density cm^−2^; AD, average diameter (mm); RV, root volume (cm^3^); SDW, shoot dry weight (mg); RL, root length (cm).

## Data Availability

Data available upon request from corresponding author.

## Supplementary Materials

The following are available online at https://www.mdpi.com/article/10.3390/ijms22137188/s1.
