# Supplementary material for: Genetic Dissection of Seedling Root System Architectural Traits in a Diverse Panel of Hexaploid Wheat through Multi-Locus Genome-Wide Association Mapping for Improving Drought Tolerance
Source: Int J Mol Sci. 2021 Jul 2;22(13):7188. doi: 10.3390/ijms22137188 (PMC8268147; doi:10.3390/ijms22137188)
Supplement: Supplementary file 1 [file ijms-22-07188-s001.zip › ijms-1267687-supplementary.pdf]

Supplementary Figures

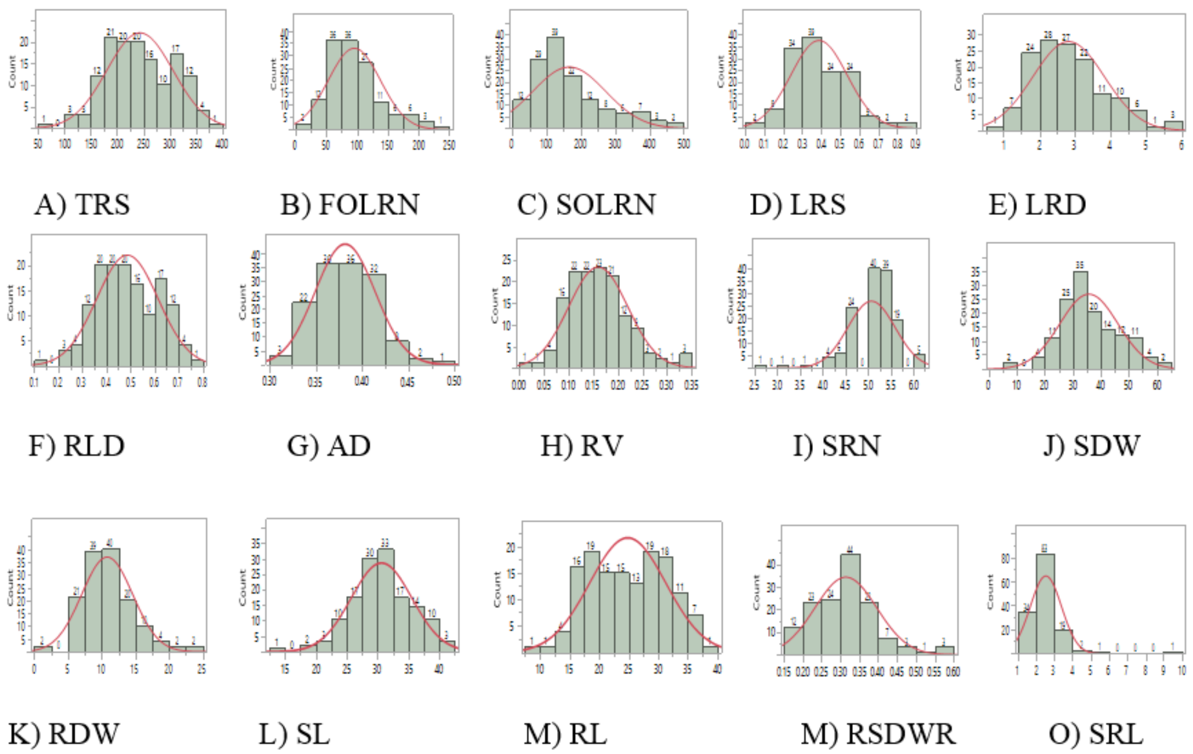

Figure S1. The frequency distribution of the phenotypic classes for root system architecture and shoot traits in wheat association panel. A) TRS, total root size (cm); B) FOLRN, first order lateral root number; C) SOLRN, second order lateral root number; D) LRS, lateral root size; E) LRD, lateral root density (cm^{-1}); F) RLD, root length density cm^{-2}; G) AD, average diameter (mm); H) RV, root volume (cm^3); I) SRN, seminal root number; J) SDW, shoot dry weight (mg); K) RDW, root dry weight (mg); L) SL, shoot length (cm); M) RL, root length (cm); N) RSDWR, root shoot dry weight ratio; O) SRL, specific root length ($cm\ mg^{-1}$).

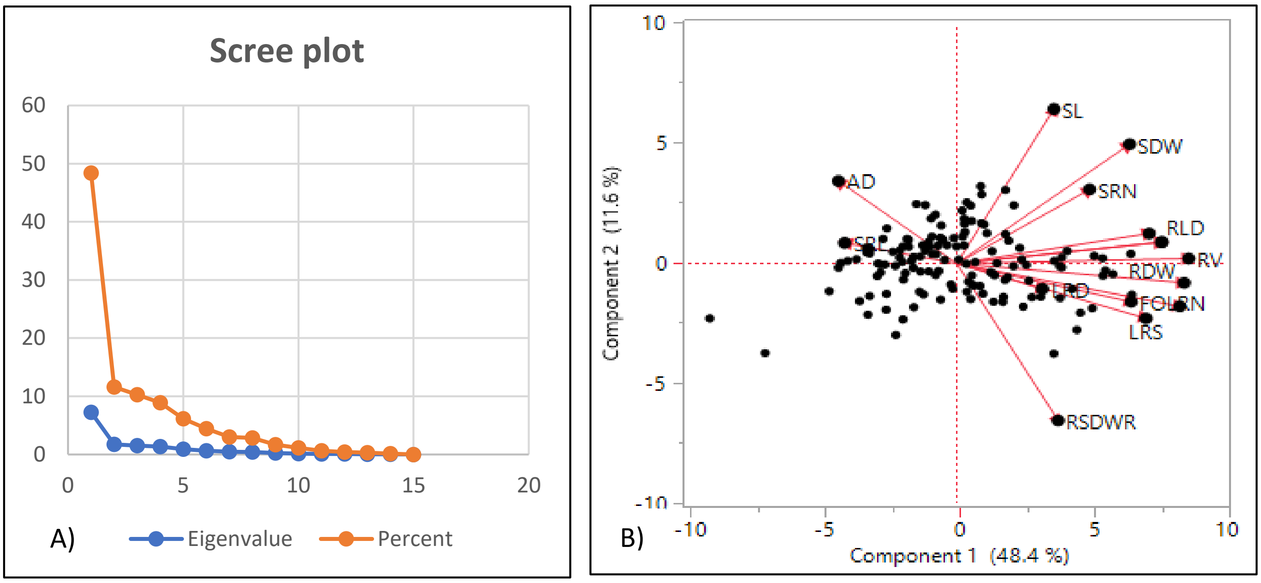

Figure S2. A) Scree plot and B) Genotype traits biplot based on principal component analysis for different root and shoot traits in wheat association panel. PCA (principal component analysis) biplots depicting the spatial distribution of genotypes as well as contribution and interrelationships between root and shoot traits.

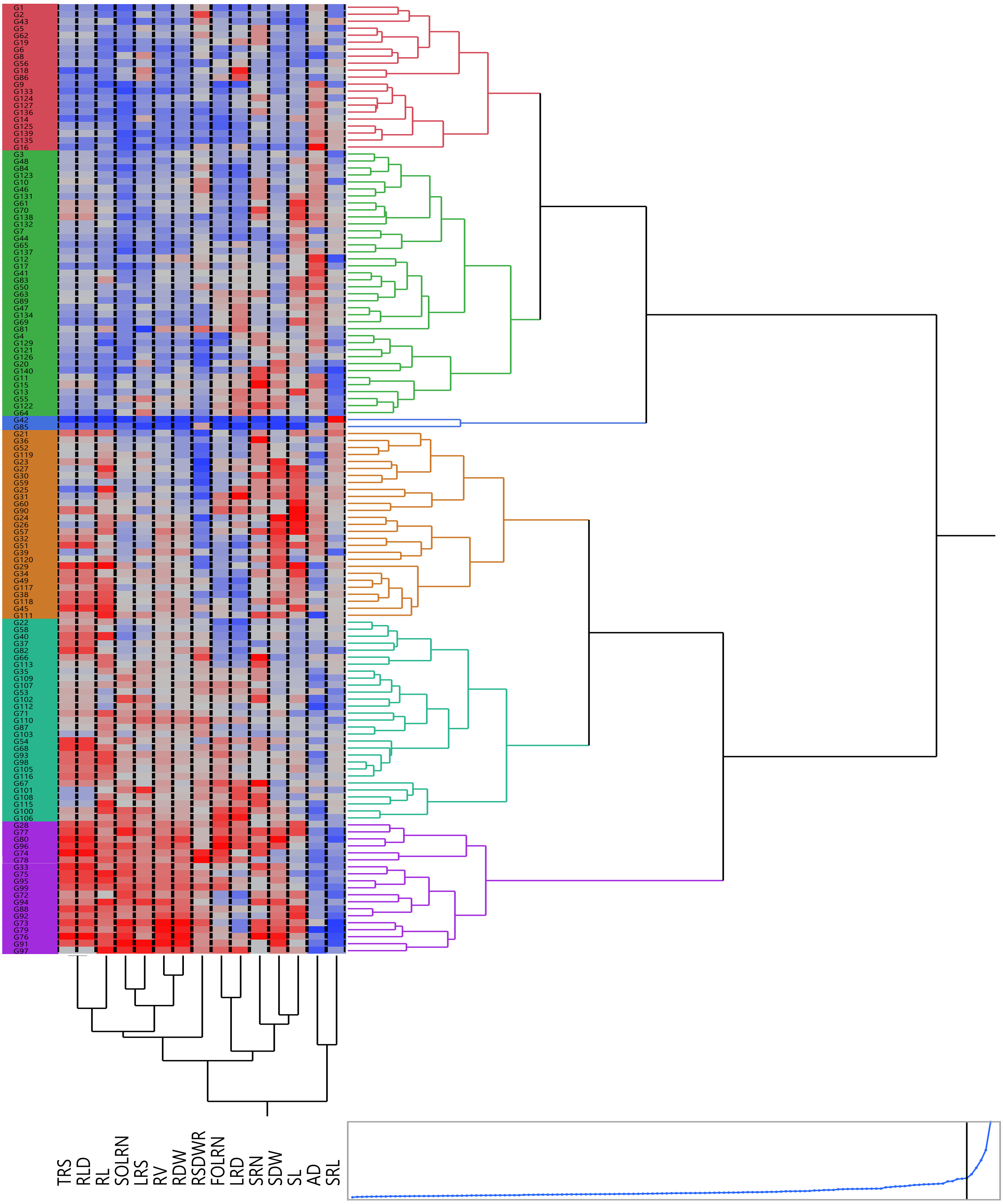

Figure S3. Dendrogram and heatmap depicting classification of genotypes based on root and shoot traits employing Euclidean distance and Ward method of clustering.

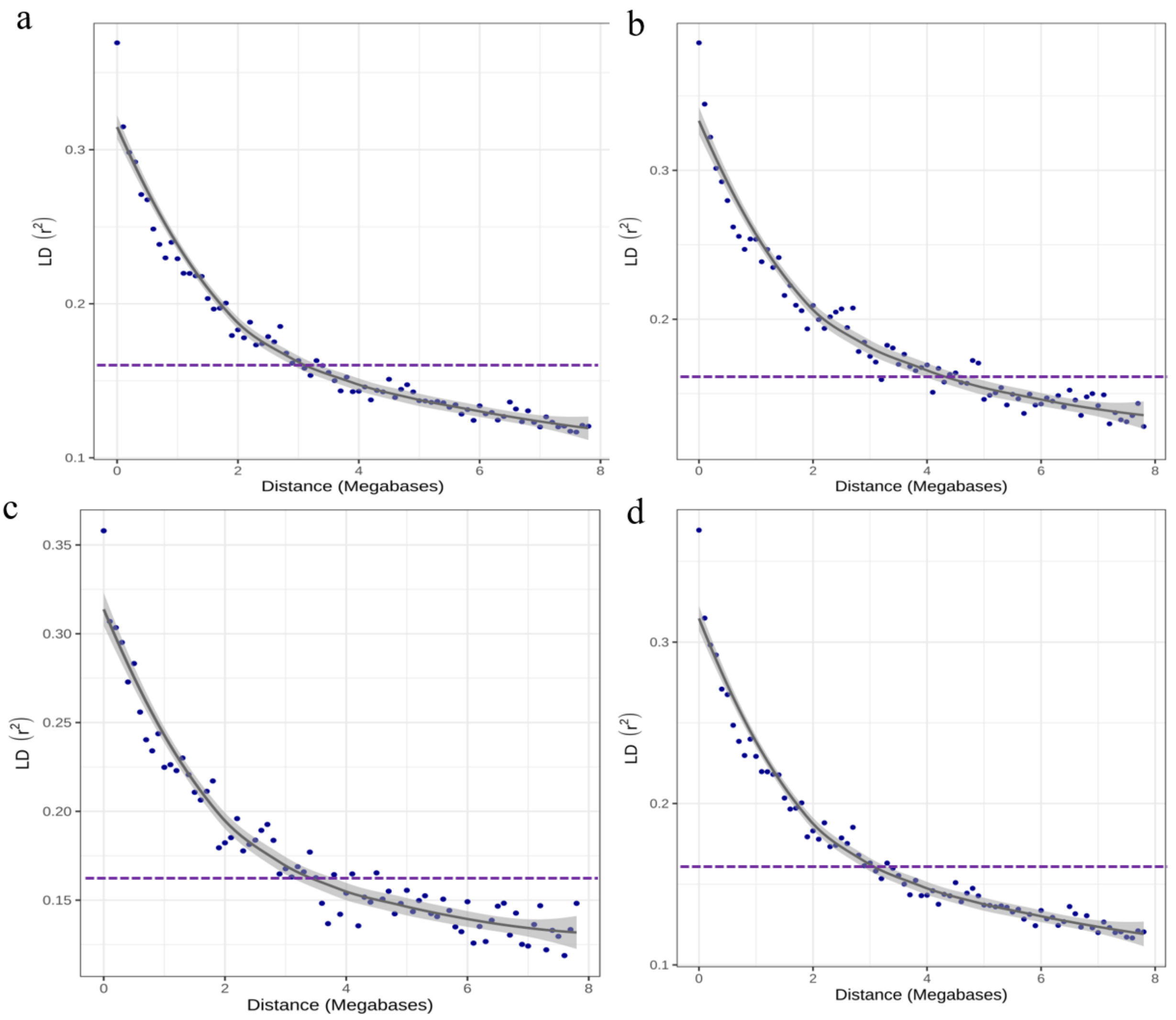

Figure S4. Linkage Disequilibrium(LD) decay plot of (a) A-genome, (b) B-genome, (c) D-genome and (d) whole-genome. Back ground LD, r^2--0.157.

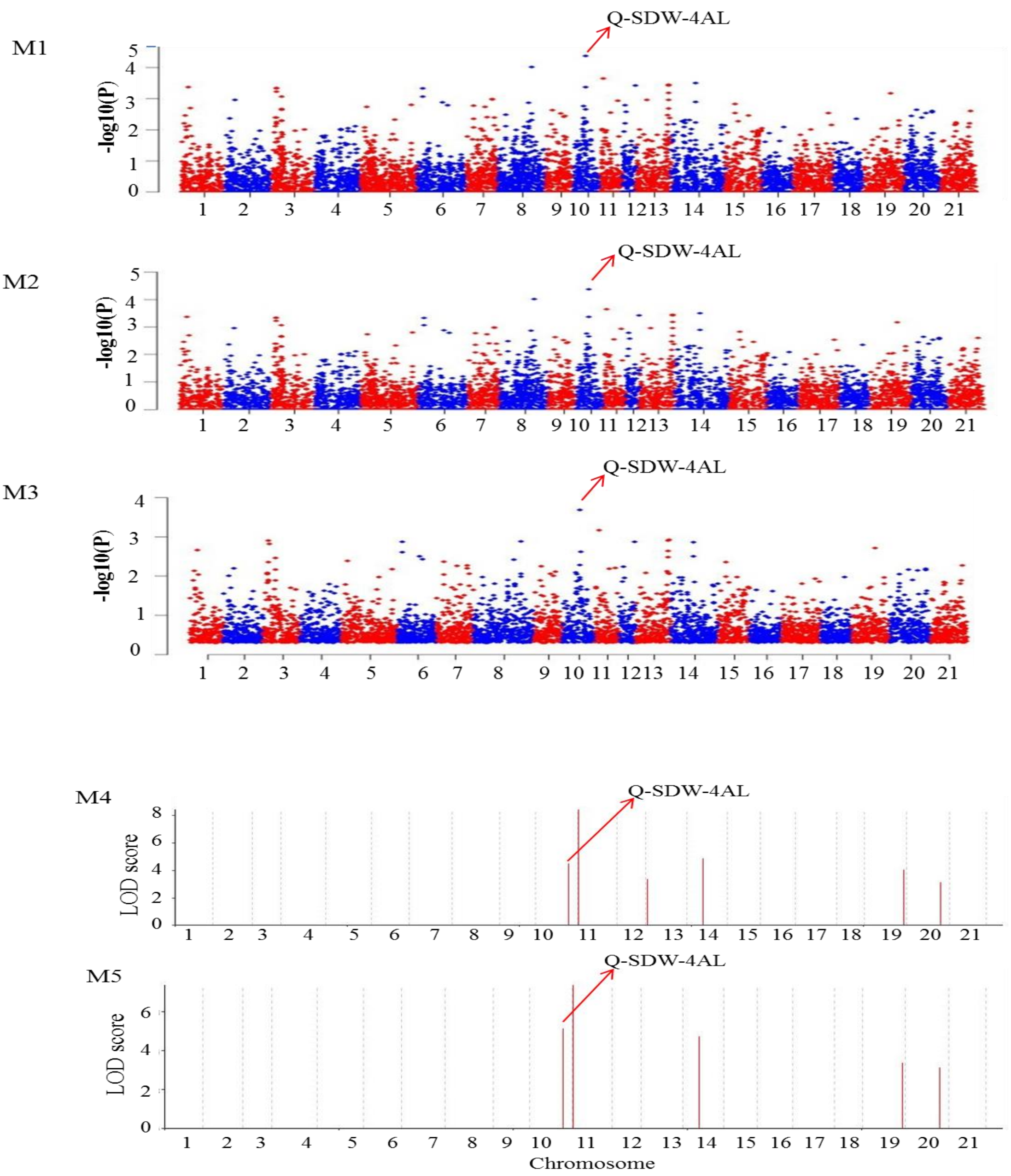

Figure S5. Manhattan plots depicting identification of a reliable quantitative trait nucleotides (QTN) for shoot dry weight, SDW (*Q.SDW-4AL*) by five multi locus genome wide association study (ML-GWAS) methods. M1: mrMLM; M2: FASTmrMLM; M3: FASTmrEMMA; M4: pLARmEB; M5: ISIS EM-BLASSO

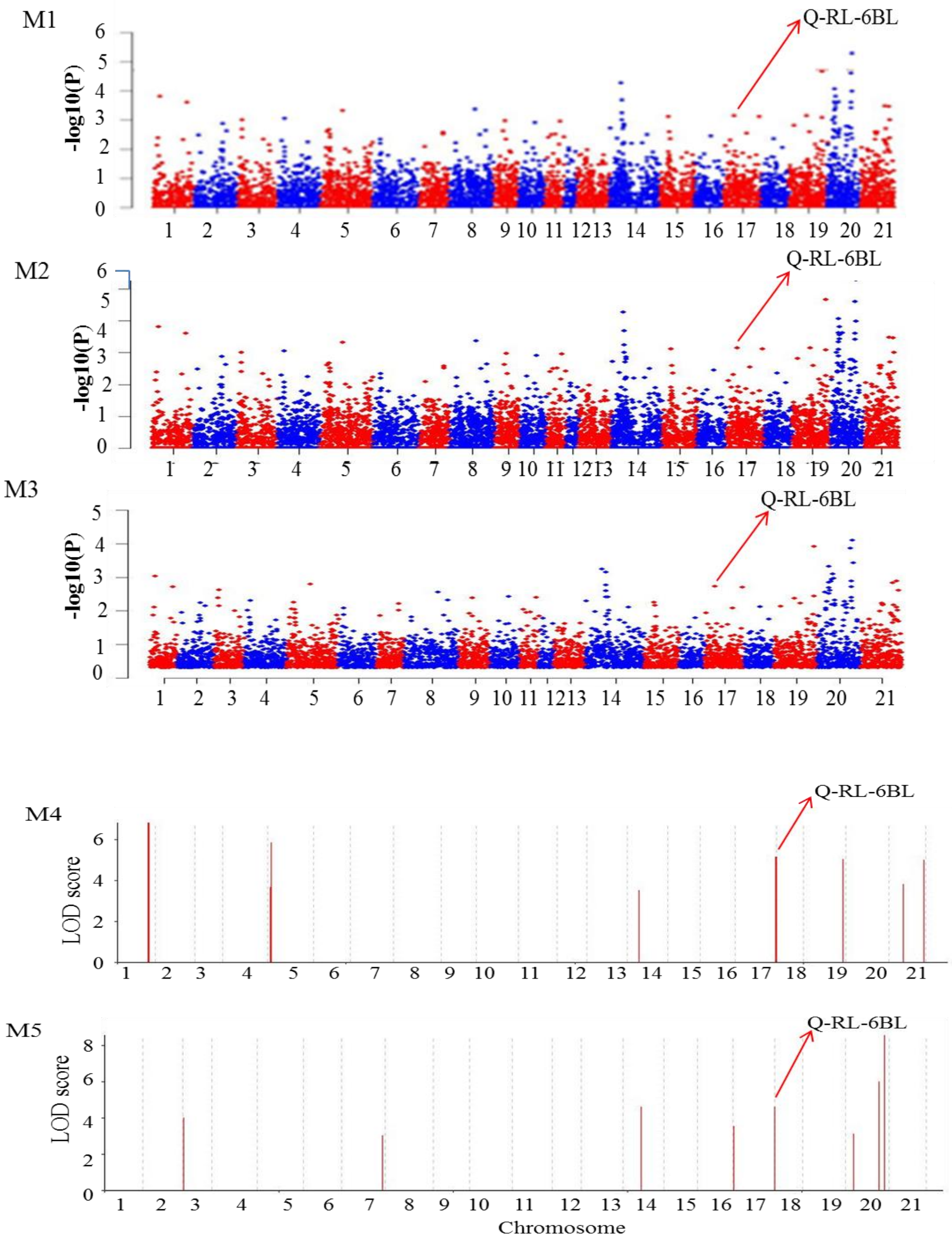

Figure S6. Manhattan plots depicting identification of a reliable quantitative trait nucleotides (QTN) for root length, RL (*Q.RL-6BL*) by five multi locus genome wide association study ML-GWAS methods. M1: mrMLM; M2: FASTmrMLM; M3: FASTmrEMMA; M4: pLARmEB; M5: ISIS EM-BLASSO.

Supplementary Tables

Table S1. Details of wheat germplasm used in the study.

S. No.	Accession	Collector No/Other ID/Cultivar Name/Pedigree	Collection Source/ Developer Institution /Country
1.	EC313710	WIR-55181, Brigantina	Ukraine
2.	IC443766	RAJ-3765 (HD-2402/VL-639)	Dungarpur, Rajasthan, India
3.	IC252796A	K-9362	CSUA&T, Kanpur, Uttar Pradesh, India
4.	IC443633	GW-1170	IIWBR, Karnal, Haryana, India
5.	IC534929	PI-348983	ICAR-NBPGR, Delhi, India
6.	EC273814	Germplasm	CIMMYT, Mexico
7.	IC533610	PI-430043	ICAR-NBPGR, Delhi, India
8.	EC313713	WIR-58801, Spartanka	USSR (Russia)
9.	EC822335A	Germplasm	CIMMYT, Mexico
10.	EC578152	SYNT-E- 51	ICAR-NBPGR, Delhi, India
11.	IC252422	BW/SH-14	West Bengal, India
12.	IC542063	MFSYCINT-166	ICAR-NBPGR, Delhi, India
13.	EC577738	E-3288	Finland
14.	EC21058	Germplasm	ICAR-NBPGR, Delhi, India
15.	IC28889	CN-92, Germplasm	Rajasthan, India
16.	EC187159	Germplasm	Israel
17.	EC574914	9084, Germplasm	ICAR-NBPGR, Delhi, India
18.	EC575981	219, Germplasm	ICAR-NBPGR, Delhi, India
19.	EC463396	Germplasm	CIMMYT, Mexico
20.	EC542279	Germplasm	USA
21.	EC576792	E-514	China
22.	EC267020	Germplasm	CIMMYT, Mexico
23.	IC26728	Germplasm	Himachal Pradesh, India
24.	IC28622	CN-47	Gujarat, India
25.	IC28029	Germplasm	ICAR-NBPGR, Delhi, India
26.	IC28872	CN-72	Rajasthan, India
27.	IC29002	Raj-234	Rajasthan, India
28.	IC28649	CN-218	Gujarat, India
29.	IC28755	A-206	Gujarat, India
30.	IC28584	Germplasm	Gujarat, India
31.	IC29008	Raj-240	Rajasthan, India
32.	IC252954	UP-2425 (HD-230/UP-2263)	Uttar Pradesh, India
33.	IC252772	J-496	ICAR-NBPGR, Delhi, India
34.	IC252469	C-578	West Bengal, India
35.	EC576816	E-563	ICAR-NBPGR, Delhi, India
36.	EC577954	E-3672	ICAR-NBPGR, Delhi, India

37.	IC582717	HPW-283	Himachal Pradesh, India
38.	IC128280	HPW-283	Himachal Pradesh, India
39.	IC252440	BW/SH-42	West Bengal, India
40.	IC574388	HD -2987	ICAR-NBPGR, Delhi, India
41.	IC252429	BW/SH-28	West Bengal, India
42.	EC339632	PI-520594	USA
43.	IC145522A	Germplasm	ICAR-NBPGR, Delhi, India
44.	EC528129A	Germplasm	ICAR-NBPGR, Delhi, India
45.	IC116274	Ajanta (BDN 519)	Maharashtra, India
46.	IC566636	PHR-1011	IIWBR, Karnal, Haryana, India
47.	IC290195	Germplasm	ICAR-NBPGR, Delhi, India
48.	EC299335	ICNO-5266	Syrian Arab Republic
49.	EC464070	CN-98	CIMMYT, Mexico
50.	IC128151	C-306	IIWBR, Karnal, Haryana, India
51.	IC282300	Halana	Uttar Pradesh, India
52.	IC252928	RW-482	ICAR-NBPGR, Delhi, India
53.	IC531183	WG- 3285	PAU, Ludhiana, Punjab, India
54.	IC252668	HP-1704	Bihar, India
55.	EC576640	E-395	NS Wales, UK
56.	EC609338	07A 37	USA
57.	IC111787	RAJ-164	Rajasthan, India
58.	IC527448	HI-1531	Madhya Pradesh, India
59.	IC335540	Kharachia-65	IIWBR, Karnal, Haryana, India
60.	IC539314	TADIA-1	IIWBR, Karnal, Haryana, India
61.	IC116276	Ajanta (BDN-519)	Maharashtra, India
62.	EC576578	E-320	Australia
63.	IC252794	K-9351	CSUA&T, Kanpur, Uttar Pradesh, India
64.	EC573974	5869	ICAR-NBPGR, Delhi, India
65.	IC531012	WL-3299	PAU, Ludhiana, Punjab, India
66.	IC554661	WH-1021	IIWBR, Karnal, Haryana, India
67.	IC406688	KCM-669	Uttarakhand, India
68.	EC576930	E-7726	USA
69.	IC290234	KRL-19	IIWBR, Karnal, Haryana, India
70.	IC335683	EGPSN(3)-71	IIWBR, Karnal, Haryana, India
71.	EC576889	E-7233	USA
72.	EC426644	Tincurrin	Australia
73.	IC449061	K-9423/Unnat Halna	CSUA&T, Kanpur, Uttar Pradesh, India
74.	IC542076	5-IAT-32	ICAR-NBPGR, Delhi, India
75.	EC574037	5952	ICAR-NBPGR, Delhi, India
76.	IC532019	Kohinoor-83	Himachal Pradesh, India

77.	IC406521	AKS/RRA/BS-171	Uttarakhand, India
78.	IC128151	C-306	IIWBR, Karnal, Haryana, India
79.	IC309875	PBW-373	PAU, Ludhiana, Punjab, India
80.	IC574476	HD-2967	ICAR-NBPGR, Delhi, India
81.	IC145237	BANSI-224	IIWBR, Karnal, Haryana, India
82.	IC542051	MIYCSN-22	ICAR-NBPGR, Delhi, India
83.	EC578153	SYNT-E- 52	ICAR-NBPGR, Delhi, India
84.	EC541159	Bruehl	USA
85.	IC303067	HW-2045 (Kaushambi) (HD2402*6//SUNSTAR*6/C-80-1)	IARI, New Delhi, India
86.	IC539313	Tadia-Genepool	IIWBR, Karnal, Haryana, India
87.	IC35163	Tadia-Genepool	IIWBR, Karnal, Haryana, India
88.	EC6903	PI-59183	USA
89.	IC82425A	Germplasm	ICAR-NBPGR, Delhi, India
90.	EC576159	E-10856	USA
91.	IC530089	VWFW-994	Uttarakhand, India
92.	IC290186	Germplasm	ICAR-NBPGR, Delhi, India
93.	IC543390	WON-D-03-51 (ET-	ICAR-NBPGR, Delhi, India
94.	IC539574	KYZ-0142	IIWBR, Karnal, Haryana, India
95.	IC329444	KVC-48	Himachal Pradesh, India
96.	EC178071-428	Germplasm	CIMMYT, Mexico
97.	IC559913A	Germplasm	ICAR-NBPGR, Delhi, India
98.	IC402044	MP-1130	IIWBR, Karnal, Haryana, India
99.	IC542040	8-EGPSN-115	ICAR-NBPGR, Delhi
100.	IC529195	VWFW-2138	Uttarakhand, India
101.	EC177789	Germplasm	CIMMYT, Mexico
102.	EC178071-454	Germplasm	CIMMYT, Mexico
103.	IC529374	VWFW-464	Uttarakhand, India
104.	IC445343	ET-96265	IIWBR, Karnal, Haryana, India
105.	IC443722	DL-803-3 (Kanchan) HUW-202/K-7537/Mutant of HD-2160	Dharwad, Karnataka, India
106.	IC415939	PAU – 355/VHL 6186	PAU, Ludhiana, Punjab, India
107.	EC463434	CN-42	CIMMYT, Mexico
108.	IC144921	GW-503	JAU, Junagarh, Gujarat, India
109.	IC335753	SAWSN (17)-33	ICAR-NBPGR, Delhi, India
110.	IC252543	GW-188	Gujarat, India
111.	IC534274	PI-168486	ICAR-NBPGR, Delhi, India
112.	IC82415	Germplasm	ICAR-NBPGR, Delhi, India
113.	IC82350	Germplasm	ICAR-NBPGR, Delhi, India
114.	EC635637	Germplasm	CIMMYT, Mexico
115.	IC533742	B-1-C-2	ICAR-NBPGR, Delhi, India
116.	EC190991	CN-67	CIMMYT, Mexico

117.	EC663921	CN-31	CIMMYT, Mexico
118.	EC635797	Germplasm	CIMMYT, Mexico
119.	EC635549	Germplasm	CIMMYT, Mexico
120.	IC78822	K-3363	CSUA&T, Kanpur, Uttar Pradesh, India
121.	IC335759	SAWSN (17)-58	ICAR-NBPGR, Delhi, India
122.	IC79041	BDJ-I-716	Himachal Pradesh, India
123.	IC252799	K-9441	CSUA&T, Kanpur Uttar Pradesh, India
124.	IC59610	Germplasm	ICAR-NBPGR, Delhi, India
125.	IC57472	Germplasm	ICAR-NBPGR, Delhi, India
126.	IC532897	WIC-455	ICAR-NBPGR, Delhi, India
127.	IC296681	Hindi-62	IIWBR, Karnal, Haryana, India
128.	IC296742	Kharachia local	Pali, Rajasthan, India
129.	EC538234	PI-631164	USDA, USA
130.	IC79058	BDJ-I-1028	Himachal Pradesh, India
131.	IC82302	BDJ-3024	Himachal Pradesh, India
132.	IC78940	K-2958	Uttar Pradesh, India
133.	IC104541	CH-5-2	Rajasthan, India
134.	IC104542	CH-6-1	Rajasthan, India
135.	IC104581	CH-42-1	Rajasthan, India
136.	IC533917	CITR-5456-A	ICAR-NBPGR, Delhi, India

Table S2. Analysis of variance (ANOVA) for studied traits under controlled condition in Completely Randomized Design.

Source of variation	Mean sum of squares	Error mean square	F value	CD@5%	CD@1%	H^2 (broad sense heritability)
DF	135	272				
TRS	12022.85	3449.3	3.48***	93.99	124.2	45.31
FOLRN	5385.91	2513.55	2.14***	80.23	106.02	27.58
SOLRN	33792.1	3596.08	9.39***	95.97	126.81	73.68
LRS	0.07	0.02	4.47***	0.20	0.26	53.64
LRD	3.02	1.97	1.53**	2.25	2.97	15.16
RLD	0.05	0.01	3.50***	0.19	0.25	45.53
AD	0.003	0.00	12.25***	0.00	0.00	78.95
RV	0.01	0.01	9.41***	0.05	0.07	73.72
SRN	0.82	0.33	2.48***	0.92	1.21	33.04
SDW	323.43	40.21	8.04***	10.15	13.41	70.13
RDW	42.92	5.31	8.08***	3.69	4.87	70.26
SL	71.71	8.33	8.61***	4.62	6.10	71.72
RL	123.25	15.95	7.72***	6.39	8.45	69.15
RSDWR	0.02	0.01	3.19***	0.12	0.16	42.3
SRL	2.21	0.47	4.67***	1.10	1.45	55.08

Significance of the difference between accessions at * $p < 0.05$. ** $p < 0.01$. *** $p < 0.001$.

DF-Degree of freedom; TRS, Total root size (cm); FOLRN, First order lateral root number; SOLRN, Second order lateral root number; LRS, Lateral root size; LRD, Lateral root density (cm^{-1}); RLD, Root length density cm^{-2}; AD, Average diameter (mm); RV, Root volume (cm^3); SRN, Seminal root number; SDW, Shoot dry weight (mg); RDW, Root dry weight (mg); SL, Shoot length (cm); RL, Root length (cm); RSDWR, Root shoot dry weight ratio; SRL, Specific root length (cm mg^{-1}).

Table S3. Top ten and bottom ten genotypes identified for root system architectural and shoot traits under pot screening (Fifteen-day-old seedlings).

S. No.	TRS	FOLRN	SOLRN	LRS	LRD
			**Top ten**		
1	IC542076 (378.62±15.01)	IC539574 (240.67±31.07)	IC82425A (490.33±83.12)	IC82425A (0.86±0.00)	IC29008 (5.89±0.44)
2	EC426644 (374.47±11.42)	IC128151 (221.00±37.50)	EC576889 (458.00±71.60)	IC329444 (0.82±0.07)	IC529374 (5.64±0.65)
3	IC128151 (361.09±11.06)	IC529374 (209.00±28.50)	EC574037 (445.67±38.17)	IC542040 (0.77±0.12)	EC575981 (5.51±0.51)
4	IC252772 (354.58±6.06)	EC426644 (202.00±11.00)	IC542076 (433.33±23.51)	IC290186 (0.75±0.12)	IC402044 (5.06±0.74)
5	IC28755 (350.10±13.52)	IC28649 (195.67±27.87)	IC329444 (428.33±43.70)	EC576889 (0.67±0.04)	IC329444 (4.90±0.87)
6	IC116274 (345.67±10.49)	IC402044 (187.67±38.61)	IC335683 (394.67±71.91)	IC559913 (0.65±0.07)	IC539574 (4.85±1.06)
7	IC531183 (344.44±10.59)	IC532019 (186.00±46.34)	IC406521 (394.67±71.91)	EC463434 (0.63±0.07)	IC443722 (4.77±1.15)
8	IC554661 (344.27±10.92)	IC559913 (178.67±26.67)	IC539574 (385.33±84.98)	EC574037 (0.60±0.02)	IC542040 (4.76±0.55)
9	IC539574 (342.33±27.96)	EC574037 (176.33±45.33)	IC449061 (379.00±45.90)	IC402044 (0.60±0.07)	IC128151 (4.64±0.46)
10	EC576889 (339.68±17.32)	EC6903 (170.67±24.33)	IC529195 (373.67±49.68)	IC543390 (0.59±0.00)	EC541159 (4.63±0.84)
			**Bottom ten**		
1	EC339632 (65.10±2.16)	EC339632 (14.33±3.18)	EC339632 (6.67±0.88)	IC309875 (0.02±0.00)	EC578153 (0.81±0.11)
2	EC575981 (108.54±13.86)	EC578153 (19.33±4.67)	EC538234 (20.00±6.00)	EC578153 (0.03±0.02)	IC267020 (1.22±0.43)
3	EC21058 (119.87±49.26)	EC822335A (29.67±4.33)	IC104581 (33.00±9.29)	EC339632 (0.11±0.03)	EC822335A (1.26±0.20)
4	EC578153 (123.44±31.69)	IC335759 (34.33±8.25)	EC822335A (35.33±2.33)	EC187159 (0.12±0.01)	EC339632 (1.26±0.20)
5	EC538234 (130.94±1.78)	IC443633 (36.67±0.67)	IC82302 (39.33±10.74)	IC104581 (0.15±0.01)	IC542051 (1.44±0.16)
6	IC28029 (138.52±17.04)	EC21058 (36.67±0.67)	IC104541 (41.00±13.32)	IC145522 (0.16±0.02)	IC128280 (1.48±0.10)
7	EC187159 (145.20±10.52)	EC538234 (44.00±12.00)	EC187159 (41.33±11.62)	EC574914 (0.18±0.07)	EC464070 (1.48±0.24)
8	IC596190 (151.39±17.51)	EC313710 (45.00±13.65)	IC542051 (43.67±13.35)	EC538234 (0.18±0.04)	EC635549 (1.48±0.05)
9	EC822335A (152.81±22.78)	IC267020 (45.67±20.54)	IC252799 (45.33±5.61)	IC104542 (0.18±0.06)	EC313710 (1.54±0.33)
10	IC252799 (154.71±19.83)	IC82302 (46.00±2.52)	EC313710 (47.67±2.40)	EC573974 (0.20±0.02)	IC335683 (1.58±0.45)
**C-1**	**(361.09±11.06)**	**(221.00±27.50)**	**(129.67±6.01)**	**(0.30±0.03)**	**(4.64±0.46)**
**C-2**	**(318.41±13.39)**	**(72.33±3.18)**	**(133.00±24.44)**	**(0.35±0.03)**	**(1.74±0.13)**

TRS, Total root size (cm); FOLRN, First order lateral root number; SOLRN, Second order lateral root number; LRS, Lateral root size; LRD, Lateral root density (cm^{-1}); C-1, Check 1 (C306); C-2, Check-2 (HD2967)

Table S3 contd… Top ten and bottom ten genotypes identified for root system architectural and shoot traits under pot screening (Fifteen-day-old seedlings).

S. No.	RLD	AD	RV	SRN	SDW
			**Top ten**		
**1**	IC542076 (0.76±0.03)	EC187159 (0.49±0.01)	EC576889 (0.35±0.02)	IC28889 (6.00±0.00)	IC128151 (60.41±5.61)
**2**	EC426644 (0.75±0.02)	IC542063 (0.47±0.01)	IC406521 (0.35±0.04)	EC577954 (6.00±0.00)	IC28622 (60.33±4.26)
**3**	IC128151 (0.72±0.02)	IC252429 (0.46±0.00)	IC82425A (0.34±0.03)	IC531012 (6.00±0.00)	IC542076 (58.61±2.35)
**4**	IC252772 (0.71±0.01)	EC574914 (0.40±0.00)	IC542076 (0.32±0.02)	IC542076 (6.00±0.00)	IC82425A (56.46±3.32)
**5**	IC28755 (0.70±0.02)	EC822335A (0.43±0.03)	EC576159 (0.28±0.03)	IC252422 (5.67±0.33)	EC609338 (56.33±2.19)
**6**	IC116274 (0.69±0.02)	IC28889 (0.43±0.01)	IC290186 (0.28±0.04)	IC28584 (5.67±0.33)	IC26728 (55.67±6.44)
**7**	IC531183 (0.69±0.02)	IC252954 (0.43±0.01)	IC329444 (0.28±0.04)	IC252772 (5.67±0.33)	IC28872 (53.00±4.04)
**8**	IC554661 (0.69±0.00)	IC35163 (0.43±0.00)	IC128151 (0.27±0.02)	EC609338 (5.67±0.33)	IC29002 (53.00±1.15)
**9**	EC576889 (0.68±0.10)	IC252799 (0.43±0.00)	IC539574 (0.26±0.05)	EC576930 (5.67±0.33)	IC290186 (52.21±4.81)
**10**	IC449061 (0.68±0.12)	IC28622 (0.42±0.01)	IC539313 (0.25±0.02)	IC406521 (5.67±0.31)	IC28755 (52.00±3.61)
			**Bottom ten**		
**1**	EC339632 (0.11±0.02)	IC406521 (0.32±0.00)	EC339632 (0.02±0.00)	EC339632 (2.67±0.33)	EC578153 (5.02±0.73)
**2**	EC575981 (0.22±0.03)	IC144921 (0.32±0.01)	EC578153 (0.03±0.01)	EC578153 (3.00±0.00)	EC339632 (5.67±0.33)
**3**	EC21058 (0.24±0.10)	IC335753 (0.32±0.00)	EC273814 (0.07±0.00)	EC187159 (3.67±0.67)	IC145522 (17.33±1.20)
**4**	EC578153 (0.25±0.06)	EC313713 (0.33±0.01)	EC575981 (0.07±0.02)	EC313710 (4.00±0.58)	EC187159 (18.56±0.12)
**5**	EC538234 (0.26±0.00)	IC28755 (0.33±0.00)	EC538234 (0.07±0.01)	IC533610 (4.00±0.00)	EC273814 (18.97±1.11)
**6**	IC28029 (0.28±0.03)	EC339632 (0.33±0.01)	IC82302 (0.07±0.02)	IC582717 (4.00±0.00)	IC443766 (19.86±4.12)
**7**	EC187159 (0.29±0.02)	IC329444 (0.33±0.01)	EC573974 (0.08±0.01)	EC528129 (4.00±0.58)	EC573974 (20.00±1.73)
**8**	EC822335A (0.30±0.05)	IC402044, (0.33±0.00)	IC78940 (0.08±0.01)	EC21058 (4.33±0.33)	EC538234 (21.10±5.82)
**9**	IC596190 (0.30±0.04)	IC533742 (0.33±0.00)	IC533917 (0.08±0.01)	EC575981 (4.33±0.67)	EC575981 (22.70±1.22)
**10**	IC82302 (0.30±0.12)	EC426644 (0.34±0.01)	EC313710 (0.09±0.02)	IC145522 (4.33±0.33)	IC104541 (22.94±1.05)
**C-1**	**(0.72±0.02)**	**(0.41±0.01)**	**(0.27±0.02)**	**(5.33±0.33)**	**(60.41±5.61)**
**C-2**	**(0.68±0.09)**	**(0.36±0.01)**	**(0.19±0.01)**	**(5.00±0.58)**	**(32.04±3.05)**

RLD, root length density cm^{-2}; AD, average diameter (mm); RV, root volume (cm^3); SRN, seminal root number; SDW, shoot dry weight (mg); C-1, check 1 (C306); C-2, check-2 (HD2967)

Table S3 contd… Top ten and bottom ten genotypes identified for root system architectural and shoot traits under pot screening (Fifteen-day-old seedlings).

S. No.	RDW	SL	RL	RSDWR	SRL
			**Top ten**		
**1**	EC576889 (23.06±1.03)	EC6903 (40.77±1.37)	IC28755 (37.50±3.40)	IC532019 (0.59±0.09)	EC339632 (9.10±1.04)
**2**	IC542076 (22.75±1.03)	IC28622 (40.67±1.72)	IC329444 (36.47±2.78)	IC252963 (0.58±0.26)	EC576792 (5.47±0.71)
**3**	IC82425A (22.17±1.27)	IC28755 (40.40±1.40)	IC449061 (36.03±2.27)	EC426644 (0.56±0.01)	EC578153 (4.26±1.53)
**4**	IC406521 (21.80±2.21)	EC609338 (39.93±1.06)	IC144921 (35.73±3.55)	IC443766 (0.50±0.16)	IC533742 (4.08±1.18)
**5**	IC128151 (19.93±1.60)	IC28872 (39.70±0.89)	IC28029 (35.40±0.55)	IC531012 (0.50±0.14)	Halana (3.95±0.82)
**6**	IC329444 (18.43±2.70)	Kharachia-65 (39.47±1.92)	IC290186 (35.33±2.19)	EC576889 (0.48±0.00)	IC29008 (3.88±1.03)
**7**	EC576159 (18.26±1.23)	IC539314 (38.60±0.40)	IC574388 (35.17±2.87)	IC559913 (0.46±0.02)	IC29002 (3.87±0.57)
**8**	IC559913 (17.80±1.94)	EC576159 (38.60±1.33)	IC116274 (35.00±1.16)	IC309875 (0.44±0.03)	IC145522 (3.75±0.88)
**9**	IC539574 (16.91±3.46)	IC29002 (37.90±1.88)	IC534274 (34.83±5.78)	EC463434 (0.44±0.01)	IC28029 (3.69±0.77)
**10**	IC539313 (16.88±0.94)	EC577738 (37.80±0.85)	IC543390 (34.50±3.68)	IC406521 (0.42±0.02)	IC28755 (3.53±0.60)
			**Bottom ten**		
**1**	EC339632 (1.07±0.04)	EC578153 (13.57±1.55)	EC339632 (9.67±0.83)	IC26728 (0.17±0.04)	EC576889 (1.31±0.07)
**2**	EC578153 (1.83±0.31)	EC339632 (18.43±0.54)	IC539464 (12.03±1.10)	IC596190 (0.17±0.00)	IC406521 (1.35±0.16)
**3**	IC82302 (5.38±1.43)	IC145522 (19.10±0.56)	EC822335A (12.57±0.43)	EC576792 (0.18±0.04)	IC539464 (1.42±0.12)
**4**	EC576792 (5.67±0.88)	EC576640 (21.67±1.95)	IC57472 (12.93±1.09)	IC29002 (0.18±0.03)	IC533917 (1.44±0.21)
**5**	EC538234 (5.85±1.40)	EC313710 (21.90±3.50)	IC252794 (13.83±0.95)	IC28622 (0.19±0.02)	IC542076 (1.45±0.06)
**6**	EC273814 (5.97±0.38)	IC443766 (22.37±1.56)	EC578153 (14.27±2.45)	IC28584 (0.19±0.00)	IC542063 (1.50±0.09)
**7**	EC576640 (6.00±0.58)	EC313713 (22.73±1.73)	EC313710 (15.10±0.71)	IC29008 (0.19±0.06)	IC82425A (1.50±0.17)
**8**	EC573974 (6.33±0.33)	EC187159 (22.73±1.02)	IC78940 (15.30±0.70)	EC339632 (0.19±0.01)	IC128151 (1.52±0.21)
**9**	IC145522 (6.43±0.81)	IC534929 (22.87±1.11)	EC463396 (15.60±0.70)	IC527448 (0.19±0.01)	IC28889 (1.63±0.16)
**10**	EC187159 (6.49±0.24)	IC449061 (22.43±0.58)	IC533917 (15.60±0.46)	IC79041 (0.19±0.00)	IC252440 (1.66±0.06)
**C-1**	**(19.93±1.60)**	**(36.90±0.40)**	**(26.83±1.01)**	**(0.22±0.01)**	**(2.38±0.22)**
**C-2**	**(13.51±1.45)**	**(25.50±1.32)**	**(25.50±1.32)**	**(0.42±0.01)**	**(1.93±0.22)**

RDW, root dry weight (mg); SL, shoot length (cm); RL, root length (cm); RSDWR, root shoot dry weight ratio; SRL, specific root length (cm mg^{-1}); C-1, check 1 (C306); C-2, check-2 (HD2967)

Table S4. Eigen value, cumulative variance (%) explained and loading value of traits for five principal components.

Traits	PC1	PC2	PC3	PC4	PC5
**TRS**	0.83	0.10	-0.41	-0.02	0.25
**FOLRN**	0.71	-0.17	0.46	0.16	0.40
**SOLRN**	0.90	-0.19	0.06	0.13	-0.14
**LRS**	0.77	-0.25	0.20	0.14	-0.41
**LRD**	0.35	-0.12	0.85	0.19	0.24
**RLD**	0.83	0.10	-0.40	-0.03	0.24
**AD**	-0.48	0.37	0.13	-0.55	0.33
**RV**	0.94	0.02	-0.11	-0.08	-0.01
**SRN**	0.54	0.34	0.20	-0.27	-0.01
**SDW**	0.70	0.54	0.07	0.07	-0.31
**RDW**	0.92	-0.09	-0.06	-0.19	-0.13
**SL**	0.39	0.70	0.14	-0.03	0.12
**RL**	0.78	0.14	-0.23	0.30	0.19
**RSDWR**	0.41	-0.71	-0.16	-0.39	0.25
**SRL**	-0.45	0.09	-0.24	0.75	0.26
**Eigenvalue**	7.26	1.74	1.55	1.34	0.93
**Cumulative variance%**	48.41	60.035	70.341	79.241	85.417

TRS, total root size (cm); FOLRN, first order lateral root number; SOLRN, second order lateral root number; LRS, lateral root size; LRD, lateral root density (cm^{-1}); RLD, root length density cm^{-2}; AD, average diameter (mm); RV, root volume (cm^3); SRN, seminal root number; SDW, shoot dry weight (mg); RDW, root dry weight (mg); SL, shoot length (cm); RL, root length (cm); RSDWR, root shoot dry weight ratio; SRL, specific root length (cm mg^{-1}).

Table S5. Clusters mean and standard deviation for root and shoot traits.

Cluster	Count		TRS	FOLRN	SOLRN	LRS	LRD	RLD	AD	RV	SRN	SDW	RDW	SL	RL	RSDWR	SRL
1	21	**Mean**	177.05	64.43	73.98	0.3	2.58	0.35	0.4	0.1	4.75	25.26	7.30	25.08	17.9	0.30	2.58
		**SD**	33.65	23.3	39.17	0.12	1.02	0.07	0.03	0.02	0.48	4.21	1.22	2.98	2.60	0.07	0.60
2	38	**Mean**	210.7	81.91	98.55	0.3	2.78	0.42	0.41	0.13	5.04	32.54	9.26	32.00	20.40	0.30	2.29
		**SD**	30.42	24.34	36.98	0.1	0.83	0.06	0.03	0.03	0.44	7.04	1.71	2.95	3.45	0.06	0.47
3	2	**Mean**	94.27	16.83	35.67	0.07	1.04	0.18	0.35	0.02	2.83	5.34	1.45	16.00	11.97	0.28	6.68
		**SD**	41.25	3.54	41.01	0.06	0.32	0.1	0.02	0.01	0.24	0.46	0.54	3.44	3.25	0.12	3.42
4	27	**Mean**	262.91	90.36	159.14	0.38	2.64	0.53	0.38	0.17	5.21	45.13	10.49	34.77	29.42	0.24	3.06
		**SD**	50.71	27.84	36.87	0.08	1.01	0.1	0.03	0.03	0.34	8.26	2.19	4.42	4.06	0.05	0.77
5	29	**Mean**	271.87	114.31	210.25	0.46	3.05	0.54	0.36	0.18	5.15	33.53	11.76	28.75	28.41	0.36	2.48
		**SD**	41.63	41.17	71.45	0.1	1.09	0.08	0.02	0.02	0.45	4.28	1.24	2.45	3.95	0.05	0.33
6	19	**Mean**	326.83	144.98	367.02	0.61	3.18	0.65	0.35	0.27	5.32	46.02	17.94	32.34	31.50	0.40	1.84
		**SD**	33.13	52.05	65.81	0.1	1.07	0.07	0.02	0.04	0.39	8.66	2.72	4.13	2.86	0.07	0.31

TRS, total root size (cm); FOLRN, first order lateral root number; SOLRN, second order lateral root number; LRS, lateral root size; LRD, lateral root density (cm^{-1}); RLD, root length density cm^{-2}; AD, average diameter (mm); RV, root volume (cm^3); SRN, seminal root number; SDW, shoot dry weight (mg); RDW, root dry weight (mg); SL, shoot length (cm); RL, root length (cm); RSDWR, root shoot dry weight ratio; SRL, specific root length ($cm\ mg^{-1}$).

Table S6. Candidate genes in the common QTN regions.

S.No.	Trait	Marker	QTNs	Position	Gene Model	Description
1	TRS, RLD, RV	AX-95105488	*Q.TRS-4AL, Q.RLD-4AL, Q.RV-4AL*	4AL: 631,901,953-631,904,813	*TraesCS4A02G358600*	RNA 3'-terminal phosphate cyclase/enolpyruvate transferase, alpha/beta
2	TRS, RLD	AX-95119337	*Q.TRS-7BS, Q.RLD-7BS*	7BS: 138877200-138885124	*TraesCS7B02G119800*	Phosphatidylinositol 3-/4-kinase, catalytic domain
3	FOLRN	AX-95249973	*Q.FOLRN-7AS*	7AS: 54997683-55001463	*TraesCS7A02G090400*	Superoxide dismutase [UniProtKB/TrEMBL;Acc:A0A1D6BYC3]
4	FOLRN, SOLRN, LRS	AX-95123855	*Q.FOLRN-7BS, Q.SOLRN-7BS, Q.LRS-7BS*	7BS: 99634205-99636250	*TraesCS7B02G086500*	Calcineurin-like phosphoesterase domain, ApaH type
5	SOLRN	AX-95077960	*Q.SOLRN-1BL*	1BL: 572296216-572302786	*TraesCS1B02G343900*	Pat1-like
6	SOLRN, RL	AX-94448890	*Q.SOLRN-1DS, Q.RL-1DS*	1DS: 10740324-10741901	*TraesCS1D02G026500*	Papain-like cysteine peptidase superfamily
7	SOLRN, RLD	AX-94516395	*Q.SOLRN-3B, Q.RLD-3B*	3B: 738693658-738699994	*TraesCS3B02G493000*	Mitochondrial carrier domain superfamily
8	SOLRN	AX-95150666	*Q.SOLRN-3B*	3B: 664018703-664023988	*TraesCS3B02G425900*	Nucleic acid-binding, OB-fold
9	SOLRN	AX-94450588	*Q.SOLRN-3DL*	3DL: 489458645-489461295	*TraesCS3A02G265000*	Histone chaperone ASF1-like superfamily
10	SOLRN, LRS, AD	AX-95244609	*Q.SOLRN-6AL, Q.LRS-6AL, Q.AD-6AL*	6AL: 599031604-599035397	*TraesCS6A02G377900*	P-loop containing nucleoside triphosphate hydrolase
11	SOLRN	AX-94564853	*Q.SOLRN-7BL*	7BL: 673955432-673963147	*TraesCS7B02G405400*	Papain-like cysteine peptidase superfamily
12	LRS	AX-94480990	*Q.LRS-3B*	3B: 417258446-417265886	*TraesCS3B02G259300*	Carbonic anhydrase superfamily
13	LRS	AX-94706358	*Q.LRS-5BS*	5BS: 69129401-69140635	*TraesCS5B02G061700*	von Willebrand factor A-like domain superfamily
14	LRD	AX-94841365	*Q.LRD-4AL*	4AL: 725751513-725752194	*TraesCS4A02G460200*	Invertase/pectin methylesterase inhibitor domain superfamily
15	LRD	AX-94571501	*Q.LRD-5DL*	5DL: 472635398-472640460	*TraesCS5D02G407700*	Protein kinase-like domain superfamily
16	RLD	AX-94544285	*Q.RLD-3DL*	3DL: 594478515-594482788	*TraesCS3D02G508100*	Protein kinase-like domain superfamily
17	RLD	AX-95659861	*Q.RLD-5AL*	5AL: 565752842-565758516	*TraesCS5A02G366100*	Potassium transporter [Source: UniProtKB/TrEMBL;Acc:A0A1D5YBR3]
18	RLD	AX-95160709	*Q.RLD-5DS*	5DS: 10468037-10472310	*TraesCS5D02G017700.1*	Modifying wall lignin-1/2
19	AD	AX-94932678	*Q.AD-1AL*	1AL: 557953578-557957082	*TraesCS1A02G390400*	UV-B-induced protein At3g17800-like
20	AD	AX-94620468	*Q.AD-1BL*	1BL: 586297635-586303171	*TraesCS1B02G357000*	ATP-dependent 6-phosphofructokinase [Source: UniProtKB/TrEMBL;Acc:A0A1D5SEJ3
21	AD	AX-94668789	*Q.AD-1BS*	1BS: 96515351-96518699	*TraesCS1B02G094600*	Nitrogen regulatory protein P-II homolog [*Arabidopsis thaliana* (AT4G01900) UniProtKB/Swiss-Prot;Acc:Q9ZST4]
22	AD	AX-94647816	*Q.AD-2DL*	2DL: 641979467-641982975	*TraesCS2D02G579600*	Protein kinase-like domain superfamily
23	AD	AX-94996868	*Q.AD-3AS*	3AS: 1028128-1031945	*TraesCS3A02G001500*	Serine-threonine/tyrosine-protein kinase, catalytic domain
24	AD	AX-94886515	*Q.AD-3B*	3B: 129436119-129441744	*TraesCS3B02G142200*	ABC transporter type 1, transmembrane domain superfamily

25	AD	AX-94726849	*Q.AD-3DS*	3DS: 81390872-81394402	*TraesCS3D02G123900*	Zinc-finger domain of monoamine-oxidase A repressor R1
26	AD	AX-94740863	*Q.AD-4AL*	4AL: 725802018-725802871	*TraesCS4A02G460400*	Invertase/pectin methylesterase inhibitor domain superfamily
27	AD	AX-94691127	*Q.AD-6BS*	6BS: 288418522-288426073	*TraesCS6B02G213500*	Protein kinase-like domain superfamily
28	AD	AX-94853162	*Q.AD-6DL*	6DL: 470293677-470298475	*TraesCS6D02G400700*	P-loop containing nucleoside triphosphate hydrolase
29	RV	AX-95162623	*Q.RV-6AL*	6AL: 611849992-611856410	*TraesCS6A02G406600*	Phosphatidylinositol-4-phosphate 5-kinase
30	RV	AX-94687596	*Q.RV-6AS*	6AS: 955213-960217	*TraesCS6A02G002500*	Translation initiation factor 3 (IF-3), N-terminal domain superfamily
31	RV	AX-94893701	*Q.RV-6AS*	6AS: 24852084-24857645	*TraesCS6A02G048800*	Glutamate receptor [UniProtKB/TrEMBL;Acc:W5GD80]
32	SRN	AX-94551988	*Q.SRN-2DL*	2DL: 579964925-579969863	*TraesCS2D02G478700*	Histone-lysine N-methyltransferase ATXR2
33	SRN	AX-94486290	*Q.SRN-3B*	3B: 477883786-477888286	*TraesCS3B02G298000*	ATPase, F1/V1/A1 complex, alpha/beta subunit, N-terminal domain superfamily
34	SRN	AX-94382595	*Q.SRN-3DL*	3DL: 392956286-392960327	*TraesCS3D02G284000*	Hexosyltransferase
35	SRN	AX-94637211	*Q.SRN-6AL*	6AL: 611575953-611577930	*TraesCS6A02G406100*	Ribosome-inactivating protein superfamily
36	SRN	AX-94790960	*Q.SRN-6AL*	6AL: 600128268-600133679	*TraesCS6A02G379300.1*	Papain-like cysteine peptidase superfamily
37	SDW	AX-94439998	*Q.SDW-3B*	3B: 130646139-130648627	*TraesCS3B02G142700.1*	F-box-like domain superfamily
38	SDW	AX-94484139	*Q.SDW-5BL*	5BL: 289562203-289567246	*TraesCS5B02G156900*	Pre-mRNA-splicing factor 38, C-terminal
39	SDW, RV	AX-94664277	*Q.SDW-7AL, Q.RV-7AL*	7AL: 689919674-689926140	*TraesCS7A02G499600.1*	P-loop containing nucleoside triphosphate hydrolase
40	SDW	AX-95165787	*Q.SDW-7BL*	7BL: 548055411-548065333	*TraesCS7D02G426800*	DNA repair protein REV1 [UniProtKB/TrEMBL;Acc:A0A1D6CPI6]
41	RDW	AX-94501549	*Q.RDW-5AL*	5AL: 672339390-672343584	*TraesCS5A02G508000*	Light-harvesting complex-like protein OHP2
42	RDW	AX-94809955	*Q.RDW-6AS*	6AS: 629428-634290	*TraesCS6A02G000800.1*	Enhancer of polycomb-like protein [UniProtKB/TrEMBL;Acc:A0A1D6ADT0]
43	SL	AX-95012217	*Q.SL-4BS*	4BS: 97921962-97925719	*TraesCS4B02G095100*	F-box-like domain superfamily
44	SL	AX-94876335	*Q.SL-7BS*	7BS: 144483417-144485194	*TraesCS7B02G123000*	UDP-glucuronosyl/UDP-glucosyltransferase
45	RL, RV	AX-95227366	*Q.RL-2BS, Q.RV-2BS*	2BS: 46088143-46091746	*TraesCS2B02G082700*	Creatinase/aminopeptidase-like
46	RL	AX-94539094	*Q.RL-6BS*	6BS: 8385071-8389993	*TraesCS6B02G013300*	P-loop containing nucleoside triphosphate hydrolase
47	RL	AX-94900754	*Q.RL-6BS*	6BS: 1963516-1970774	*TraesCS6B02G002500*	Protein kinase-like domain superfamily
48	RL, LRS	AX-94528392	*Q.RL-7BL, Q.LRS-7BL*	7BL: 675313213-675319653	*TraesCS7B02G406700*	P-loop containing nucleoside triphosphate hydrolase
49	RL	AX-94861078	*Q.RL-7DL*	7DL: 614275351-614277978	*TraesCS7D02G512800*	Cytochrome P450 superfamily
50	RSDWR	AX-94856412	*Q.RSDWR-2BL*	2BL: 754863037-754865182	*TraesCS2B02G562500*	Protein kinase-like domain superfamily
51	SRL	AX-94457792	*Q.SRL-2BL*	2BL: 576080648-576091559	*TraesCS2B02G406800*	Formin-like protein
52	SRL	AX-94748697	*Q.SRL-5AL*	5AL: 406534556-406537319	*TraesCS5A02G200700.1*	Succinate dehydrogenase subunit 7, mitochondrial

TRS, total root size (cm); FOLRN, first order lateral root number; SOLRN, second order lateral root number; LRS, lateral root size; LRD, lateral root density (cm^{-1}); RLD, root length density cm^{-2}; AD, average diameter (mm); RV, root volume (cm^3); SRN, seminal root number; SDW, shoot dry weight (mg); RDW, root dry weight (mg); SL, shoot length (cm); RL, root length (cm); RSDWR, root shoot dry weight ratio; SRL, specific root length ($cm\ mg^{-1}$).
